# Parallel evolution of Bacteroidota as long-term endosymbionts of insects

**DOI:** 10.1093/ismejo/wrag259

**Published:** 2026-10-09

**Authors:** Jinyeong Choi, Cong Liu, Pradeep Palanichamy, Yumiko Masukagami, Hirotaka Tanaka, Takumasa Kondo, Matthew E Gruwell, Filip Husnik

**Affiliations:** Evolution, Cell Biology, and Symbiosis Unit, Okinawa Institute of Science and Technology Graduate University, Okinawa 904-0495, Japan; Evolutionary Genomics Unit, Okinawa Institute of Science and Technology Graduate University, Okinawa 904-0495, Japan; Evolution, Cell Biology, and Symbiosis Unit, Okinawa Institute of Science and Technology Graduate University, Okinawa 904-0495, Japan; Evolution, Cell Biology, and Symbiosis Unit, Okinawa Institute of Science and Technology Graduate University, Okinawa 904-0495, Japan; Faculty of Agriculture, Ehime University, Tarumi 3-5-7, Matsuyama, Ehime 790-8566, Japan; The Kyushu University Museum, Hakozaki 6-10-1, Higashi-ku, Fukuoka 812-8581, Japan; Corporación Colombiana de Investigación Agropecuaria-Agrosavia, Centro de Investigación Palmira, Palmira, Valle del Cauca 763531, Colombia; Penn State Erie, Behrend College, School of Science, Erie, PA 16563, United States; Evolution, Cell Biology, and Symbiosis Unit, Okinawa Institute of Science and Technology Graduate University, Okinawa 904-0495, Japan

**Keywords:** genome reduction, symbiotic organ, scale insects, flavobacteriales

## Abstract

Symbiotic relationships significantly transform both symbiont and host biology. The most visible changes include a massive reduction of the endosymbiont genome and the development of specialized host organs, cells, and compartments for symbiont housing. However, insect symbiosis studies have often focused on Pseudomonadota or specific symbiosis stages, limiting our understanding of how symbiont diversity arises from particular microbial clades, how the symbiont genomes evolve, and the consequences of symbiosis for diverse host-symbiont pairs. Scale insects, which have experienced repeated gains and losses of nutritional symbionts, provide an ideal evolutionary playground for tracking parallel transitions of insect symbionts originating from the same bacterial phylum. Using extensive genome sampling across the scale insect phylogeny, we recapitulated the path of Bacteroidota transitioning from recently established host-associated bacteria to highly specialized insect endosymbionts with minimal gene sets. Their genomes exhibit strikingly parallel degradation across all gene categories, with some stochastic differences in essential genes for genetic processing and amino acid biosynthesis. Imaging methods further revealed that the symbiotic cells and organs exhibit a trend from more dispersed bacteriocytes to highly compact bacteriomes, closely localized to the host’s digestive system. Our results outline a recurrent path by which insect symbioses independently arise, are maintained for up to several hundred million years, and are replaced by new symbionts. The gradual nature of the process implies that the outcomes of symbiosis initially depend on how “professional” vs. “naïve” the host and symbiont are. Over evolutionary time, compatible host-symbiont lineages emerge from these interactions and become preferred.

## Introduction

The evolutionary fates of microbial symbionts often converge on similar outcomes, and for maternally transmitted symbionts, these trajectories become nearly irreversible due to population-genetic processes [1]. With ongoing gene loss due to relaxed selection and random genetic drift accelerated by intra-host localization and bottlenecks during transmission [2], most symbiont genes are eliminated over time. Genes that are erased last are involved in DNA replication, transcription, and translation, as well as in a few pathways that confer benefits to the host [3, 4]. This further pseudogenization of the important symbiont genes is caused by accidental early stop codons arising from nonsense mutations [5]. To alleviate this disruption in protein-coding genes, the old symbiont genomes have occasionally undergone codon reassignments, resulting in an alternative genetic code that translates the UGA codon to tryptophan instead of its original role as a translational stop signal [6]. In many cases, the symbiont functions have been shown to be replaced or complemented by host genes of both foreign and native origin or by additional coobligate symbionts [7–10]. However, despite these processes slowing down symbiont degradation, most long-term endosymbionts face extinction, replacement by new microbial partners, or, in some cases, organelle-like integration [11,12].

Bacteroidota play critical roles in nutrient cycling in both aquatic and terrestrial ecosystems [13]. Many Bacteroidota are also crucial symbionts of animals, plants, and other eukaryotes. Their diverse ecological niches and multifunctional roles are reflected in their high genomic diversity and plasticity [13]. In insects, Bacteroidota are well known as beneficial symbionts that establish long-term evolutionary relationships with diverse insect clades [4,14–17]. These relationships have been proposed to expand the ecological niches of the host insect groups and promote their diversification [18]. A textbook example is *Candidatus* Karelsulcia, which has been maintained by auchenorrhynchan insects for ~300 million years, and its strains exhibit a substantial genome reduction (142–285 kb) [14,19,20]. In terms of genome size, *Karelsulcia* is followed by *Candidatus* Shikimatogenerans (172–200 kb) and *Candidatus* Bostrichicola (323–346 kb) in bostrichid beetles [4], *Blattabacterium* (511–645 kb) in cockroaches and termites [21,22], and *Candidatus* Skilesia (1.32 Mb) in aphids [16]. However, available sampling of Bacteroidota symbionts in insects is limited to only one or two lineages per insect group, and only when combined, they represent multiple stages of genome reduction, highlighting the importance of broader sampling specific to a single insect clade.

Scale insects house long-term obligate symbionts that provide them with essential amino acids and vitamins lacking in their plant sap diets [23,24]. In contrast to other insect clades with Bacteroidota symbionts, scale insects have a dynamic evolutionary history with various lineages of Bacteroidota symbionts [23,25,26]. Despite the diversity of Bacteroidota lineages recognized as associated with scale insects, genome sequencing and microscopy have been limited to only three genera of Bacteroidota symbionts, *Candidatus* Walczuchella (270–309 kb) [27,28], *Candidatus* Uzinuria (263 kb) [29,30], and *Candidatus* Insignibacter (866 kb) [31]. Here, we analyzed 21 genomes of 10 different Bacteroidota lineages from diverse scale insect clades. Our paired host-symbiont phylogenomic analyses robustly show that scale insects have acquired Bacteroidota symbionts multiple times independently. We reveal significant variation in the symbiont genome sizes and gene contents, suggesting ongoing pseudogenization and gene loss that depend on the age of the symbiont. Additionally, we show with microscopy that, depending on the symbiont genome reduction level, the localization of symbiont-harboring cells and organs tends to shift from a widespread distribution throughout the host body to a more centralized localization near the insect gut. These results contribute to understanding the evolutionary dynamics of Bacteroidota, showing how they frequently transition into highly integrated symbionts of insects and suggesting how compatible host–symbiont lineages evolve over hundreds of millions of years.

## Materials & methods

### DNA extraction and metagenome sequencing

Metagenomes of 18 species were newly generated in this study. Total genomic DNA was extracted with the MasterPure Complete DNA Purification Kit (Epicenter) and DNeasy Blood and Tissue Kit (Qiagen) following the manufacturer’s protocols. PCR-free sequencing libraries were prepared with the NEBNext Ultra II DNA library preparation kit. In addition to newly generated data, the Sequence Read Archive (SRA) data from 23 species were reused in this study. In total, we analysed metagenomes from 41 species in 16 families of scale insects (Supplementary Table S1). Paired-end sequencing was performed on the Illumina NovaSeq 6000 sequencer at the Okinawa Institute of Science and Technology Graduate University and Illumina MiSeq at SEQme and Penn State University. We confirmed the quality of raw sequencing reads using FastQC v0.11 [32] and filtered out low-quality reads with fastp v0.20 [33]. The taxonomic affiliation and relative abundance of different organisms were profiled using phyloFlash v.3.4 [34] based on 16S and 18S rRNA gene sequences from the metagenomes.

### Phylogenetic analyses of symbionts

Phylogenetic trees of symbionts were reconstructed using the rRNA gene sequences and single-copy orthologous genes. The rRNA gene phylogeny was primarily used to identify symbionts from metagenomes. The 16S/18S rRNA and 23S/28S rRNA gene sequences of bacterial and fungal symbionts were extracted from metagenome assemblies with Barrnap v3 (https://github.com/tseemann/barrnap). Additionally, we also used published genome assemblies and rRNA sequences of related symbionts and free-living bacteria from NCBI. The 16S/18S rRNA and 23S/28S rRNA gene sequences were concatenated after individual alignment with MAFFT v.7 [35] and trimming with trimAL v.1.5 [36] (−automated1). We predicted orthologous genes using OrthoFinder v.2.5.4 [37] and extracted only 65 genes that were present as single-copy in all the Bacteroidota genomes. These protein sequences were aligned with MAFFT v.7 (−auto), trimmed with trimAL v.1.4 (−automated1), and then concatenated. Additionally, we sampled single-copy orthologous genes from genome assemblies of Bacteroidota with BUSCO v.5.1.3 [38] (with the bacteroidetes_odb10). The protein sequences of 118 orthologous genes were aligned with MAFFT v.7 and concatenated with Seqkit [39] after trimming each gene sequence with trimAL v.1.4 (−automated1). Maximum likelihood analyses for all the sequence matrices above were performed with IQ-TREE v.1.6.9 [40]. The substitution models were chosen with ModelFinder [41] implemented in IQ-TREE. Node support was assessed with 1000 ultrafast bootstrap replicates. The phylogenetic trees were visualized using Figtree v.1.4.4 [42]. The following Candidatus status names have been assigned to Bacteroidota lineages and one *Sodalis*-like cosymbiont that currently lack a scientific description. First, *Candidatus* Brownia xenocola is for the symbionts of *Eumyrmococcus scorpioides*. The species name *xenocola* refers to the host family, Xenococcidae. This bacterium belongs to the same genus as *Brownia rhizoecola*, a symbiont of Rhizoecidae, due to their close phylogenetic relationship. Second, *Candidatus* Conchaspibacter symbioticus is for the symbionts of *Conchaspis* sp. Third, *Candidatus* Orthebacter symbioticus is for the symbionts of *Orthezia urticae*. Fourth, *Candidatus* Acanthobacter symbioticus is for the symbionts of *Acanthococcus lagerstroemiae*. Fifth, *Candidatus* Paraacanthobacter symbioticus is for the symbionts of *Acanthococcus* sp. The genus names are derived from the associated hosts, with “-bacter” indicating their bacterial affiliation, whereas the “symbioticus” species names reflect their symbiotic lifestyle within the hosts. Last, *Candidatus Sodalis rastrocola* is for the cosymbionts of *Rastrococcus iceryoides*. The species name *rastrocola* refers to the host genus, *Rastrococcus*.

### Symbiont genome assembly and annotation

To obtain the draft genomes of Bacteroidota symbionts and one *Sodalis*-like cosymbiont, the raw Illumina reads were assembled using SPAdes v.3.15 [43] with specific k-mer sets and options (Supplementary Table 2). In some cases, we used the merged reads generated by PEAR v.0.9.11 [44] and the untrusted-contigs option with reference genomes. The assembled sequences were taxonomically assigned using megablast search (NCBI-BLAST v.2.11.0) against the NCBI nucleotide database (−culling_limit 5, −evalue 1e-25). The taxon-annotated-GC-coverage plots were generated for each metagenome with Blobtools v.1.1.1 [45]. Based on these plots, the genome assemblies of Bateroidota symbionts were extracted from the metagenomes. Due to the high number of contigs of the *Orthebacter* genome, we used MetaBAT2 v.2.12.1 [46] to bin the genome and assessed its completeness with the DASTool v.1.1.6 [47]. Genome assemblies with 2 or 3 contigs were closed manually using the alignment with other assemblies and assembly graphs as a guide. The joint regions of the original contigs were confirmed by read mapping and visualization with Tablet v.1.21.02.08 [48]. *Acanthobacter* and *Conchaspibacter* genomes were assembled based on their PacBio reads using Flye v.2.9 (−meta) [49]. Assembly graphs of all the Bacteroidota genomes were visually assessed with Bandage v.0.9 [50]. All the genome assemblies of Bacteroidota were polished using Pilon v.1.24 [51]. PacBio-only assemblies were polished with Illumina short reads available from NCBI. Genome annotations were carried out with Prokka v.1.14.6 [52]. Some gene predictions were confirmed with Bakta v.1.8.1 [53] and GhostKOALA v.2.0 [54]. Bacteroidota symbiont RAIC was found to be using an alternative genetic code, so we used the genetic code 4 (UGA > Trp) for gene prediction instead of the code 11 used for all the other symbiont genomes. Hypothetical proteins were re-annotated through BLASTp searches against the NCBI RefSeq (NR) database [55]. The transfer RNA genes (tRNAs) were predicted with tRNAScan-SE v.2.0 [56]. Missing intact CDSs in the gene prediction were recovered with BLASTx searches of intergenic regions, implemented in Pseudofinder v.1.1.0 [57]. The coding sequences (CDSs) were classified into clusters of orthologous groups (COGs) using eggNOG-mapper v.2 [58] with default parameters. Orthologs of Bacteroidota genomes were inferred with OrthoFinder v.2.5.4 [37]. Genome feature maps were created with DNAPlotter [59]. A synteny plot of Bacteroidota genomes was generated with Processing3 (https://processing.org) based on tBLASTx genome alignments and Prokka annotations.

### Pseudogene and selection pressure predictions

The putative pseudogenes of Bacteroidota symbionts were predicted with PseudoFinder v.1.1.0 with DIAMOND v.2.0.4.142 [60] searches against NR. We further estimated the selective pressure on the symbiont genes mostly involved in genetic information processing and amino acid (AA) biosynthesis. The nonsynonymous mutation rate to the synonymous mutation rate (*dN/dS*) of each orthologous gene was calculated using the yn00 program based on pairwise comparisons in PAML v.4.9 [61]. The free-living bacterium, *Flavobacterium johnsoniae* (GCF_034479105), with nonreduced genomes (> 6 Mbp), was used as a standard for the sequence comparison. The orthologous gene pairs of each Bacteroidota symbiont and the free-living bacterium were identified using OrthoFinder. For genes with paralogs, we selected only one sequence of each bacterium based on its sequence similarity. The AA sequences of orthologous genes were aligned with MAFFT v.7 (−auto). The codon-based DNA alignment was obtained with PAL2NAL v.14 [62] based on the aligned AA sequences and unaligned nucleotide sequences. We considered the genes with a *dN*/*dS* ratio ranging from 0.1 to 0.95 to be evolving under relaxed purifying selection [63].

### Host genome assemblies and phylogenomic analyses

A custom pipeline was built to extract the host insect genomes from 39 metagenome assemblies. First, we removed contigs shorter than 400 bp and mapped the raw reads onto the filtered metagenomic assembly with minimap2 [64]. Then we computed the sequencing depth, GC content, and coding density for each contig with SprayNPray v.1 [65]. We also searched the contigs against the nonredundant (nr) database with DIAMOND v.2.0.13.151 [66] and assigned a putative phylum to each contig with MEGAN v.6.24.21 [67] if available. Second, we trained a decision tree classifier using scikit-learn, with the sequencing depth, GC content, and coding density as training features and phylum as the target value. This classifier inferred phyla for contigs that were not classified by MEGAN. Finally, we extracted contigs assigned to Arthropoda and manually removed contigs with distinct depth and GC content from the main cluster. The resulting insect genome bins were further polished using FCS-GX [68]. The polished genome assemblies evaluated by QUAST [69] and BUSCO (with the hemiptera_odb10). A total of 57 species were used for phylogenomic analysis, including 18 published genomes retrieved from NCBI. A matrix of concatenated protein sequences was assembled from 359 single-copy genes identified by BUSCO v.5.1.3 following the methods as described above for the symbiont phylogenetic analysis. A maximum likelihood (ML) tree was produced with IQ-TREE under the best-fitting model (JTT + F + R6) selected automatically, and 1000 ultrafast bootstrap replicates were performed to assess the node support. Divergence times were estimated using MCMCTree in PAML v.4.9 [61] with the independent rate model. The gradient and Hessian required for MCMCtree dating were calculated using IQ2MC [70] in IQ-TREE. Eight fossil calibrations, representing the oldest known fossils of the corresponding clades, were used as minimum-age constraints [71] (Supplementary Table 3). The root age was constrained to <300 million years ago (Mya) according to the fossil information and previous study [72,73]. We performed two independent runs (nsample = 10 000, sampfreq = 5000, burnin = 2 000 000) to confirm the convergence of MCMC chains with Tracer v.1.7.2 [74]. The mean and 95% confidence intervals were assessed. All the phylogenetic trees were visualized with FigTree.

### Microscopy

The spatial distribution of Bacteroidota symbionts in host insects was examined to investigate changes across different stages of symbiosis, using an integrated approach of fluorescence in situ hybridization (FISH) and micro-computed tomography (μCT). We analyzed three host species (ACLA, RASP, and RAIC), harboring Bacteroidota symbionts at early, intermediate, and late stages of symbiosis based on their genome status (Figs 2 and 3). Symbiont localization in other host species was inferred from previous histological studies. For FISH, the distribution of symbionts was confirmed using a 16S rRNA probe CFB319 (Cy3-TGGTCCGTGTCTCAGTAC), targeting Bacteroidota [14]. Whole insect tissues were fixed for 1 day in 4% paraformaldehyde in phosphate-buffered saline (PBS-1×) and Carnoy’s solution. After washing with 80% ethanol, tissues were bleached for 2–4 weeks in 6% hydrogen peroxide solution, which was replaced with fresh solution several times. After washing with absolute ethanol, the bleached tissues were embedded in Technovit 8100 resin (Kuzler, Wehereim, Germany) and sectioned into 3–5 μm sections using a rotary microtome (HM 340E, Epredia). The sections were incubated overnight at room temperature in hybridization buffer containing DAPI (1 μg/μl) and probes (100 nM). The samples were mounted with ProLong Diamond Antifade Mountant (Thermo Fisher Scientific) after washing with PBS. Tissues were then examined under the Nikon ECLIPSE Ti2-E (Nikon, Tokyo, Japan) inverted microscope. The three-dimensional distribution of symbionts was further confirmed using μCT. The samples were stained with iodine for 1–3 days and scanned using the Zeiss XRadia Versa410 X-ray microscope, operated with the Zeiss Scout-and-Scan Control System software (v.14.0). Scanning parameters included a beam strength of 40 kV and 3 W power, with exposure time of 13–19 s under 4× objective lens, resulting in voxel size of 1.09–1.49 μm. Post processing was performed using Amira 2019.2 software (Visage Imaging GmbH, Berlin, Germany).

## Results

### Well-supported scale insect phylogeny with divergence time estimates

Draft genomes of 39 species of scale insects were newly assembled in this study (Supplementary Table 4). The average size and GC content of the assemblies, including 18 NCBI references, were 365 Mbp and 33%, respectively. The average scaffold N50 of assemblies was 17 Mbp, and 41 genomes had N50 above 10 Kbp. The BUSCO completeness of 57 genomes averaged 75%, with 38 genomes exhibiting completeness above 80%. Maximum likelihood analysis with IQ-TREE was well-resolved for most clades (Fig. 1; Supplementary Fig. 1). The Coccomorpha clade was estimated to have originated ~328 Mya (Fig. 1; Supplementary Fig. 2). Among hosts with long-term Bacteroidota symbionts, Monophlebidae, Rhizoecidae + Xenococcidae, and Diaspidiae species formed distinct clades. First, the Monophlebidae clade was estimated to have appeared ~164 Mya in the Jurassic. Second, the Rhizoecidae + Xenococcidae clade was estimated to have originated 127 Mya in the Cretaceous. Third, the Diaspididae clade was estimated to have originated 67 Mya in the Paleogene or possibly the upper Cretaceous.

**Figure 1:**
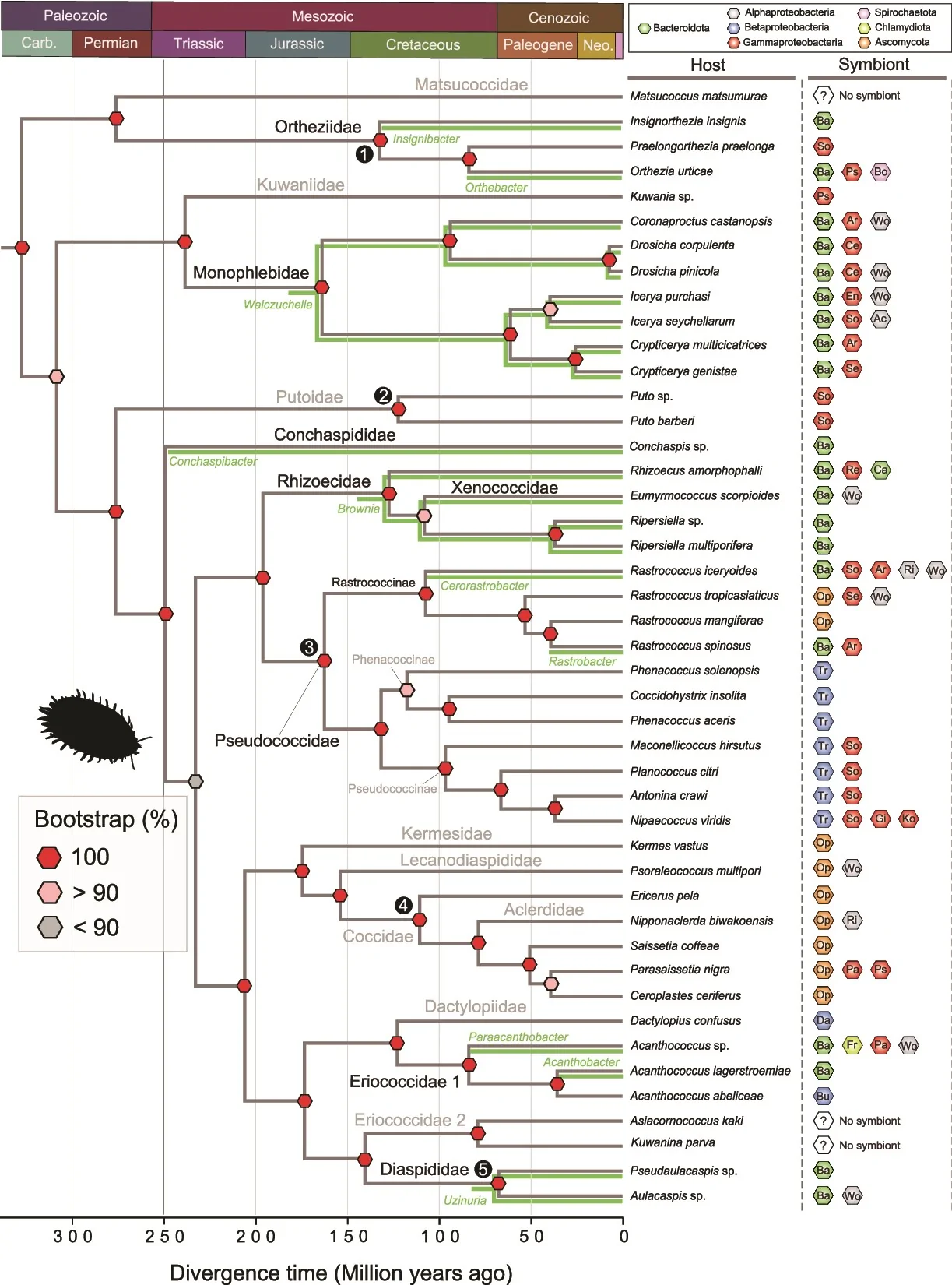
Time-calibrated phylogenetic tree of scale insect hosts and their microbial symbionts. The divergence times were estimated by MCMCtree under the independent-rate model with eight fossil calibrations, five of which are denoted by numbered black circles in the ingroup. Ultrafast bootstraps estimated by IQ-TREE are shown for each node using three colors (red: 100%; pink: > 90%; gray: < 90%). Detected microbial symbionts are represented by specific symbols next to the corresponding host species labels. Symbiont taxonomic abbreviations are as follows: Ac: Acetobacteraceae, Ar: *Arsenophonus*, Ba: Bacteroidota, Bo: *Borrelia*, Bu: *Burkholderia*, Ca: *Cardinium*, Ce: *Cedeceae*, Da: *Dactylopiibacterium*, En: *Enterobacter*, Fr: *Fritschea*, Gi: *Gilliamella*, Ko: *Kosakonia*, Op: *Ophiocordyceps*, Pa: *Pantoea*, Ps: *Pseudomonas*, Re: *Regiella*, Ri: *Rickettsia*, Se: *Serratia*, So: *Sodalis*, Tr: *Tremblaya*, Wo: *Wolbachia*. Branches representing host species with Bacteroidota symbionts are highlighted with shaded green lines.

### Scale insects show extreme symbiont diversity with multiple lineages of Bacteroidota symbionts

Diverse bacterial and fungal symbionts were detected from scale insects (Fig. 1). Most metagenomes (49%) included one major symbiont with a high sequence coverage and 1–4 additional co-symbionts. The remaining metagenomes contained a single symbiont (44%) or lacked a dominant symbiont (7%). Most symbionts were bacteria from the phyla Bacteroidota and Pseudomonadota (Supplementary Fig. 3). Some symbionts were fungi that mostly fell within the clade of *Ophiocordyceps* in Ascomycota (Supplementary Fig. 4). Our phylogenetic analyses support at least 10 independent origins of Bacteroidota becoming long-term endosymbionts of scale insects (Figs 1 and 2). All these Bacteroidota were clustered in a distinct clade with other insect symbionts within the Flavobacteriales clade (Supplementary Fig. 3). In the two phylogenomic analyses that are largely congruent (Supplementary Fig. 5), most Bacteroidota symbionts of scale insects were nested within a single clade including *Skilesia*, except for *Brownia* and *Cerorastrobacter* in a separate clade with the other insect symbionts. In the cophylogenetic analysis, overall topologies were not mirrored between symbionts and hosts (Fig. 2; Supplementary Fig. 6). Only Bacteroidota symbionts of Diaspididae, Monophlebidae, and Rhizoecidae showed congruent topologies with their hosts.

**Figure 2:**
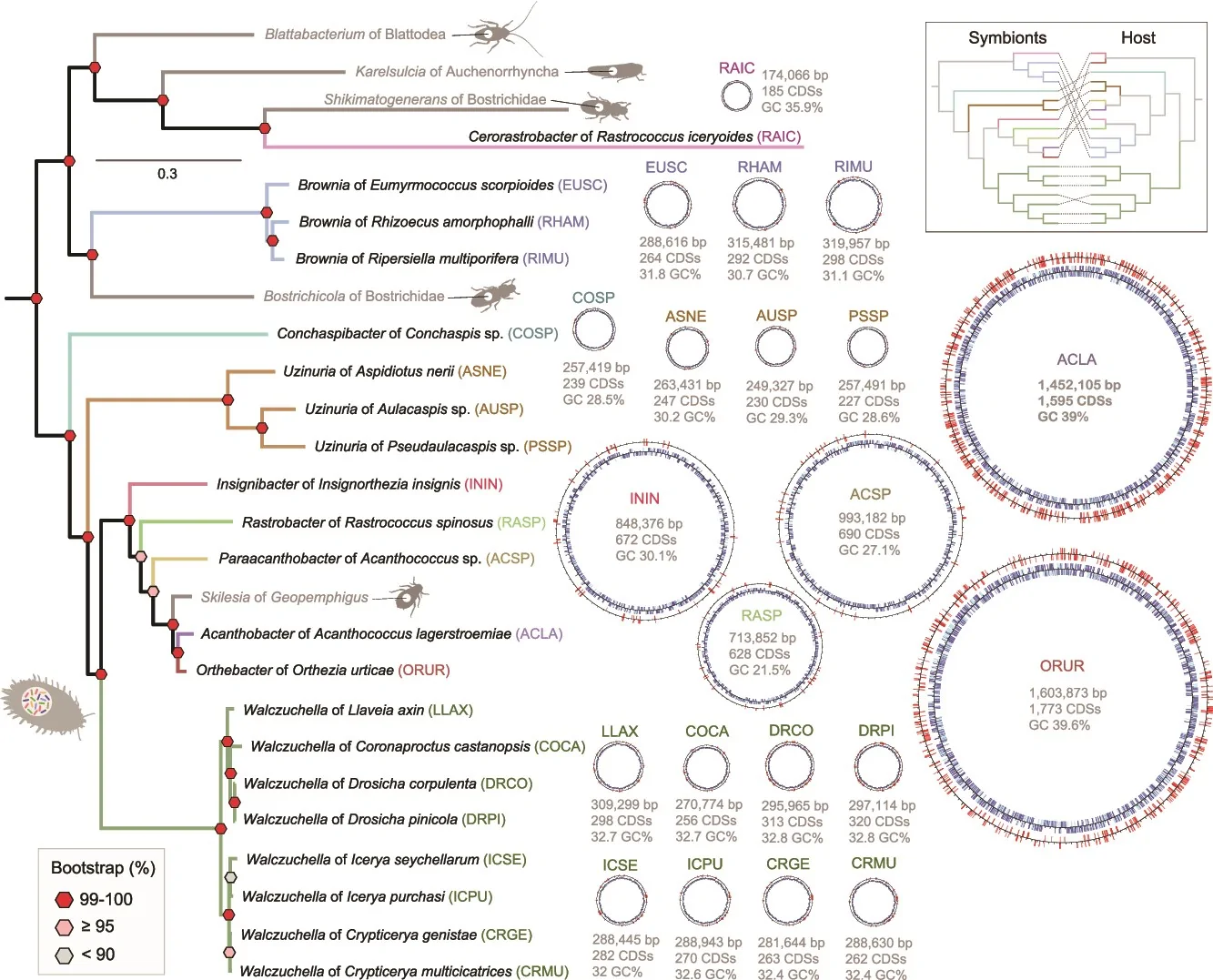
Phylogenetic relationships of Bacteroidota symbionts from scale insects with their genome features. The maximum likelihood tree was inferred with IQ-TREE based on 118 BUSCO genes under the JTTDCMut+F + R5 substitution model. Ultrafast bootstraps are displayed for each node using three different colors (red: 99–100%; pink: ≥ 95%; gray: < 90%). Tip labels of symbionts from scale insects are shown in black, whereas those of symbionts from other insects, including aphids, auchenorrhynchans, bostrichids, and cockroaches, are shown in gray. Genome maps of 21 Bacteroidota symbionts from scale insects are placed alongside the phylogenetic tree. In the genome maps, intact genes (blue) and pseudogenes (red) are represented on the inner and outer circles, respectively. The cophylogeny of symbionts and their hosts is shown on the top right.

### Multiple independent origins of the long-term Bacteroidota symbionts are reflected both in their genomes and localization

We analyzed a total of 21 genomes of Bacteroidota symbionts from scale insects, most of which were closed as complete circular-mapping genomes (Figs 2 and 3; Supplementary Table 2). The genome size of the Bacteroidota symbionts varied greatly (174–1604 kb). The number of protein-coding genes (CDSs) of the scale insect symbionts correlated with their genome sizes, from 170 to 1773, and coding densities from 70% to 95%. The smaller symbiont genomes (170–340 Kbp) showed a higher coding density of over 79%. One exception was *Rastrobacter* with an intermediate genome size (714 kb) but a high coding density of 88%. The genomes have GC content ranging from 21.5% to 39.6%, which does not correlate well with their genome sizes (Fig. 3). CDSs predicted as putative pseudogenes ranged between 2–27%, with the genomes of *Walczuchella* (10–27%), *Orthebacter* (26%), and *Acanthobacter* (27%) being the most highly pseudogenized. Putative plasmids were found in *Insignibacter* and *Conchaspibacter*. Likely due to their independent origins, Bacteroidota genomes were highly rearranged and shared only limited blocks of synteny (Supplementary Fig. 7). The spatial distribution of Bacteriodota symbionts is summarized in Supplementary Table 5. *Orthebacter* and *Acanthobacter*, with larger genomes, are primarily distributed throughout the host body and are associated with fat body cells, as shown by FISH imaging (Fig. 4A). *Insignibacter* and *Rastrobacter*, possessing an intermediate-sized genomes, are intracellularly localized within host cells (Fig. 4B). These bacteriocytes are distributed throughout the body or restricted to region beneath the ventral host membrane. *Cerorastrobacter* is also intracellular, forming a compact organ (Fig. 4C). This bacteriome is centrally located in the host’s abdomen, near the midgut.

**Figure 3:**
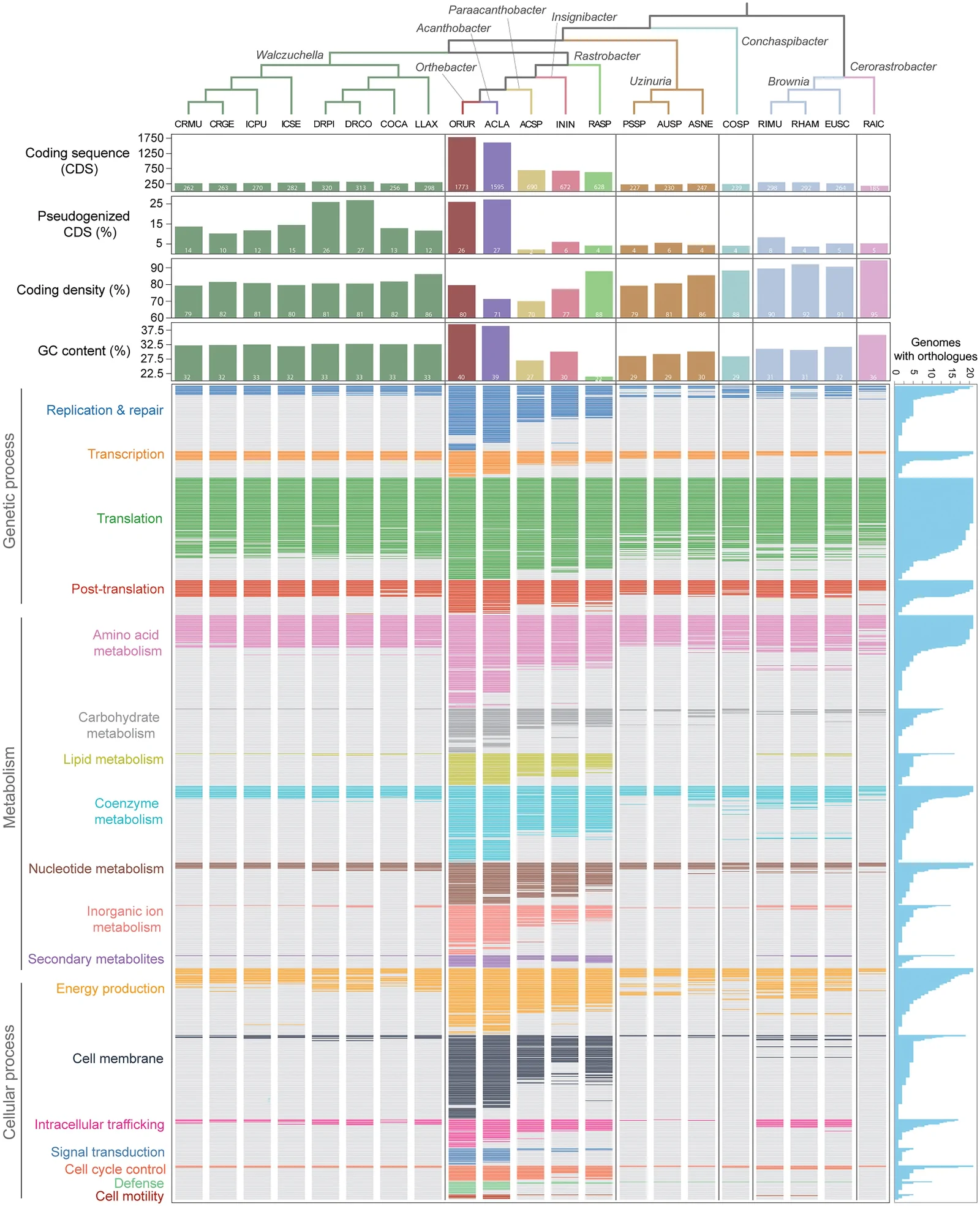
Genome properties and functional gene contents of Bacteroidota symbionts from scale insects. The bar plots indicating basic genome features are displayed above the corresponding gene content figure. Orthogroups (orthologous genes) defined with OrthoFinder were sorted into COGs by eggNOG-mapper. Colored boxes represent the presence of orthologous genes, except for pale gray color representing the absence of related genes in the genomes. The functional gene categories are listed on the left side of the main figure. The bar plot on the right indicates the number of genomes retaining corresponding orthologous genes. The phylogenetic tree represents the relationship of the symbionts.

**Figure 4:**
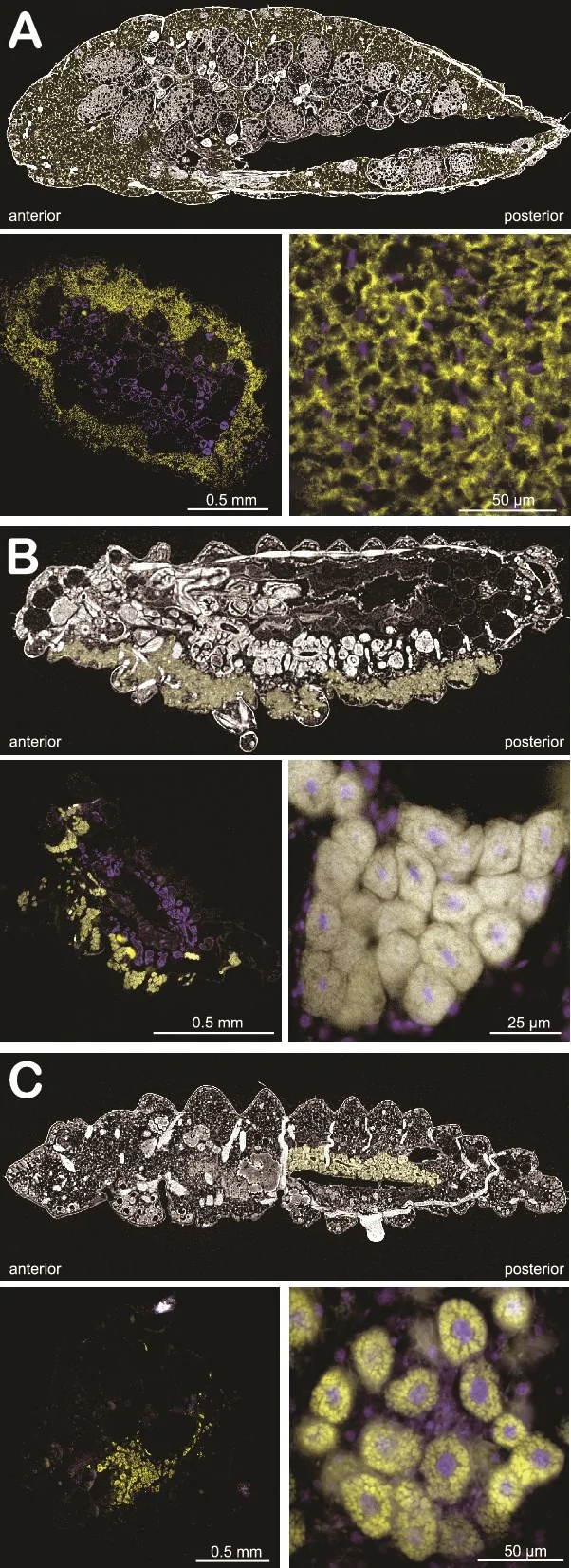
Localization of Bacteroidota in scale insects. (A) *Acanthococcus largerstromae*, (B) *Rastrococcus spinosus*, and (C) *R. iceryoides*. Each panel includes body sections visualized by micro-computed tomography (μCT) (top) and fluorescence in situ hybridization (FISH) (bottom). The FISH images show whole-body sections (left) and magnified symbiont-associated host cells (right). In the μCT images, symbiont-associated cells are highlighted in yellow. In the FISH images, Bacteroidota symbionts are shown in yellow and host cell nuclei in blue.

### Functional gene categories lost rapidly vs. more gradually

Massive gene loss has occurred in all the functional gene categories of the analyzed genomes (Fig. 3). The large symbiont genomes (with 700*–*1600 kb, hereafter, LSGs) retain 54–94% of the total orthogroups identified from all the Bacteroidota genomes. However, smaller symbiont genomes (with <320 kb, hereafter, SSGs) showed much lower retention rates of orthogroups (17–28%). Although gene loss of Bacteroidota symbionts generally occurred in all the COGs, the protein-coding genes for translation (J), post-translational modifications (O), and AA metabolism (E) categories were proportionally increased in the SSGs compared to the LSGs (Supplementary Fig. 8).

#### Only 114 genes are conserved across all the Bacteroidota symbionts of scale insects

We report 114 protein-coding genes that are retained in all the Bacteroidota symbionts of scale insects, with their *dN/dS* (ω) and predicted pseudogenes (Fig. 5A; Supplementary Table 6). Most of these conserved genes are related to genetic information processing (75%) and AA biosynthesis (20%). The average ω for all 114 genes was close to 0.15, excluding pseudogenes. The gene encoding 30S ribosomal subunit S20 (*rpsT*) represented the highest average ω of 0.46 among the conserved genes. Among symbionts, *Cerorastrobacter* showed the highest average ω of 0.28. The genes for DNA polymerase (*dnaE*) and RNA polymerase (*rpoABCD*) were intact in most symbiont genomes with 0.09–0.2 average ω values. Among translation-related genes, translation factors (*infABC*, *fusA, tuf*, and *tsf*) were conserved but *infC* of the two LSGs showed pseudogenization. Other conserved genes were mostly for ribosomal proteins, aminoacyl-tRNA synthetases, and post-translational processes.

**Figure 5:**
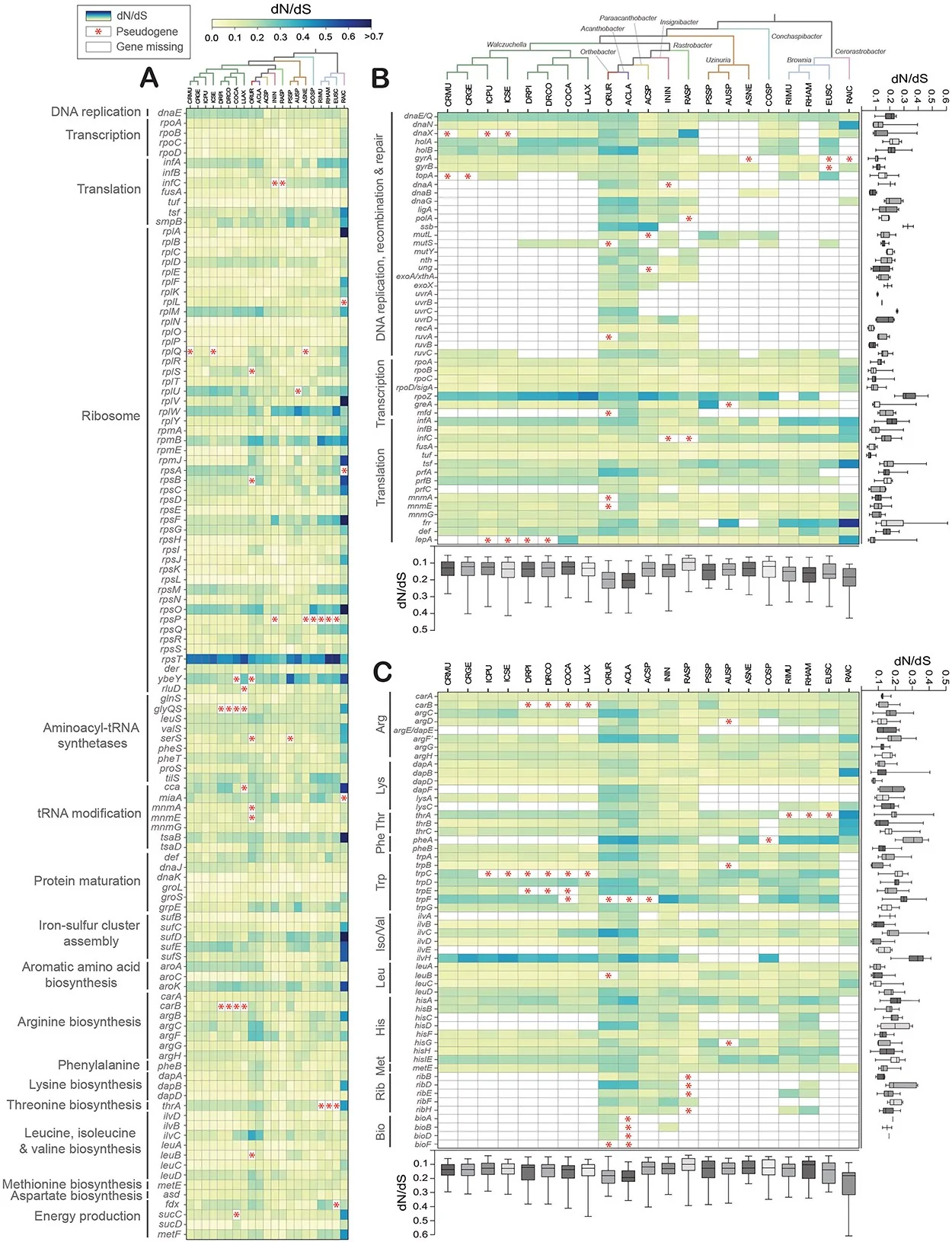
Retention pattern for selected genes of Bacteroidota symbionts from scale insects. (A) 114 conserved genes of the symbionts; important genes for the genetic processes (B), and amino acid and vitamin metabolism (C). Color-coding represents ratios of nonsynonymous to synonymous substitutions (*dN/dS*; ω) of the retained genes. The *dN/dS* values for each retained gene across symbionts and for all retained genes within each symbiont are also shown as box plots on the right side and below the main figure, respectively. The genes flagged as pseudogenes by PseudoFinder are indicated with red asterisks (*). The missing genes are represented in white. The gene names and their related functions are listed on the left, with standard abbreviations for amino acids and B vitamins. The phylogenetic trees represent the relationships of the symbionts.

#### Even genes that are generally conserved in other bacteria are lost from some symbiont genomes

We thoroughly examined the gene loss patterns in the genetic machinery and nutrition-related genes (Figs 5B-C; Supplementary Tables 7 and 8). The SSGs lost many key genes for DNA replication, and DNA recombination and repair genes. In the LSGs, some of these genes were also lost or pseudogenized. Among transcription-related genes, only the RNA polymerase subunit ω (*rpoZ*), transcription elongation factor (*greA*), and transcription-repair coupling factor (*mfd*) are missing or pseudogenized in some SSGs. The termination factor encoding gene (*prfC*) was lost from all the SSGs, as well as *Insignibacter* (LSG). In addition, *prfA* and/or *prfB* were not detected in *Brownia* EUSC and *Cerorastrobacter* genomes. The SSGs showed loss and pseudogenization of at least one gene for each AA biosynthesis. *Cerorastrobacter* lost all important genes for cysteine, tryptophan, and histidine biosynthesis, which are likely complemented by the cosymbiont *Sodalis rastrocola* (Fig. 6). This symbiont possesses the smallest genome among currently known *Sodalis-*allied symbionts. Additionally, the riboflavin and biotin biosynthesis genes are missing in all the SSGs, except for a few retentions of riboflavin genes in some *Brownia* genomes. The biotin genes were mostly missing in the LSGs as well as the SSGs. In the genetic machinery and nutritional gene categories, *Acanthobacter*, *Orthebacter*, and *Cerorastrobacter* have higher average ω from 0.19 to 0.24, compared to the overall averages of 0.16 and 0.15 for these categories.

**Figure 6:**
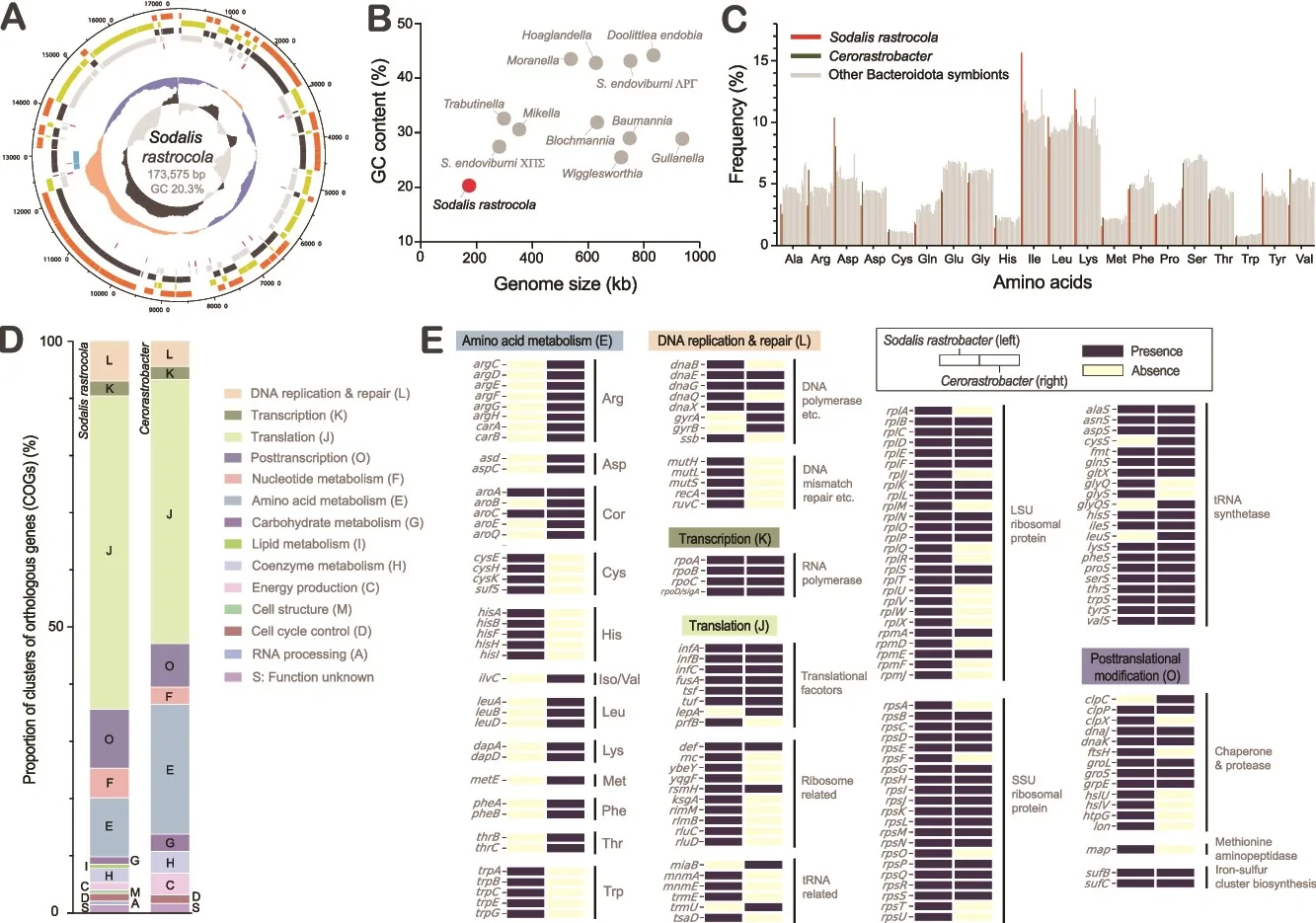
Genome properties of *Sodalis rastrocola* and comparison with *Cerorastrobacter*. (A) Genome map of *S. rastrocola*. From the outermost to innermost rings: (i) CDSs on the forward strand (orange), (ii) CDSs on the reverse strand (yellow), (iii) genes on the forward strand (dark gray), (iv) genes on the reverse strand (light gray), (v) tRNA genes (purple), (vi) rRNA genes (blue), (vii) GC content (above average, pale orange; below average, blue), and (viii) GC skew (above average, dark gray; below average, light gray). (B) Genome size and GC content of known *Sodalis*-allied insect symbionts. (C) Amino acid composition of translated CDSs in symbiont genomes. Both *S. rastrocola* and *Cerorastrobacter* show relatively low frequencies of cysteine, histidine, and tryptophan in their predicted proteomes compared to other amino acids. (D) Comparison of the proportion of clusters of orthologous groups (COGs). *S. rastrocola* retains approximately half as many genes involved in amino acid metabolism as *Cerorastrobacter*, but substantially more genes related to genetic processes.

### *Cerorastrobacter* uses an alternative genetic code and lost all protein release factors

The tiny genome of *Cerorastrobacter* was inferred to use an alternative genetic code. The gene prediction for this genome with the genetic code 11 (UGA > STOP) resulted in highly fragmented protein-coding genes, in contrast to genetic code 4 (UGA > Trp) (Fig. 7A). This difference was caused by pervasive in-frame UGA codons in the open reading frames. In the genome annotation with the genetic code 4, at least one in-frame UGA codon was found in 55% of CDSs (Table 1). We also confirmed that the *Cerorastrobacter* genome has the UGA codon at the conserved positions of the *dnaE* and *valS* genes encoding tryptophan in the other symbiont genomes (Fig. 7B). The proportion of UGG encoding tryptophan was only 0.5% of the total codons across all its protein–CDS, which is lower than 0.7–1.0% reported from endosymbiont genomes with the genetic code 11 (Fig. 7C). Alternatively, the UGA codon, which also encodes tryptophan, accounted for 0.3% of the codons in the Bacteroidota RAIC genome. The anticodon of tryptophan tRNA (*trnW*) is UCA in *Cerorastrobacter*, which contrasts with the other symbionts having CCA as its anticodon. The UAA and UAG represent 83% and 17% of the total stop codons in the genome of *Cerorastrobacter* (Fig. 7D). This UAA proportion was much higher than the average (64%) in the other symbionts. *Cerorastrobacter* does not contain any genes encoding peptide release factors recognizing UAA and UAG as stop codons. Except for *Brownia* EUSC, all the other Bacteroidota symbionts retain both *prfA* and *prfB* in their genomes (Fig. 5B).

**Figure 7:**
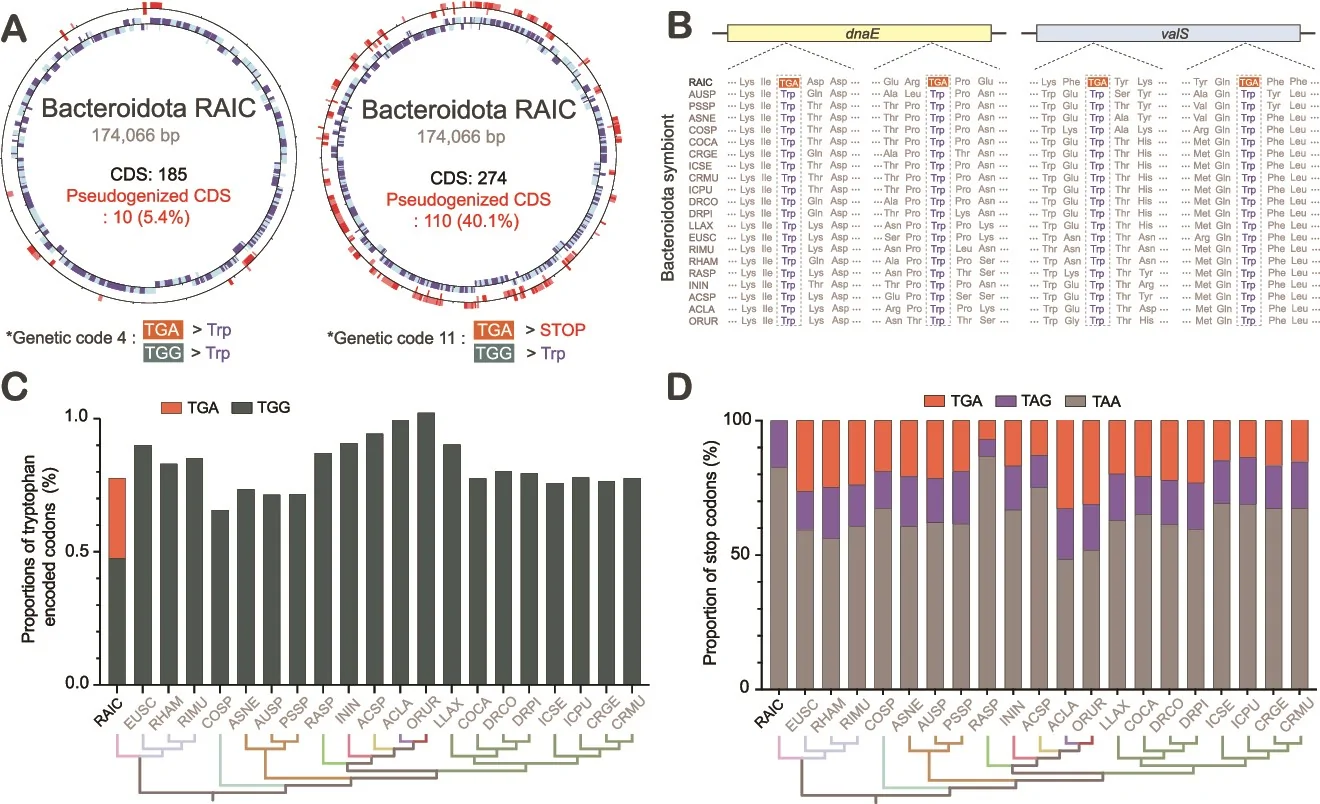
Genome features of *Cerorastrobacter* using an alternative genetic code. (A) Comparison of genome annotations using genetic codes 4 and 11 showing differences in the numbers of protein-coding genes (CDS) and pseudogenized CDSs. (B) Conserved positions encoding tryptophan in the *dnaE* and *valS* genes of Bacteroidota symbionts from scale insects. Proportions of tryptophan-encoding codons (C) and stop codons (D) in the symbiont CDSs. The phylogenetic trees indicate the relationships of the symbionts.

**Table 1:** Features of select animal symbionts using the genetic code with UGA: stop - > tryptophan.

Class	Flavobacteriia	Alphaproteobacteria	Betaproteobacteria		Gammaproteobacteria	
Symbiont	*Cerorastrobacter*	*Hodgkinia*	*Nasuia*	*Zinderia*	*Stammera*	*Providencia*
*prfA*	−	+	+	+	+	+
*prfB*	−	−	−	−	−	+(ψ)^a^
*trnW* anticodon	UCA	UCA	UCA	CCA	CCA	CCA
GC%	35.8	58.4	17.1	13.5	15.4	22.0
CDSs	185	169	137	206	255	610
In-frame UGA (%)	101 (55%)	119 (70%)	69 (50%)	121 (59%)	159 (62%)	125 (21%)

a Including a UGA stop codon in the *prfB* pseudogenes.

## Discussion

### Repeated acquisitions, losses, and replacements have shaped Bacteroidota symbioses in scale insects

Our results revealed a complex evolutionary history of interactions between microbial symbionts and scale insect hosts (Fig. 1). We identified 10 different lineages of Bacteroidota that have established symbiotic relationships with eight scale insect families. Because only five well-studied long-term Bacteroidota symbionts were previously known from other insect lineages, our work expands the known diversity of insect-associated Bacteroidota to 15 lineages. Based on our time-calibrated scale insect phylogeny, several Bacteroidota symbioses have been maintained for tens to hundreds of millions of years. For example, *Brownia*, *Uzinuria*, and *Walczuchella* likely entered into symbiosis with their respective hosts ~60–160 million years ago (Fig. 1). *Cerorastrobacter* and *Conchaspibacter* may also have similarly ancient symbioses with their hosts based on their genome reduction levels (Fig. 2). Although these associations appear to be much younger than the ancient symbiosis between Auchenorrhyncha and *Karelsulcia*, which has persisted for ~260–300 million years [14,75], scale insects exhibit a more dynamic evolutionary history of association with Bacteroidota symbionts.

Our findings indicate that independent acquisitions of Bacteroidota occurred in multiple scale insect clades, followed by prolonged host-symbiont codiversification or symbiont loss and replacement by other bacteria and fungi (Fig. 1). Although the ancient *Karelsulcia* symbiont has also been lost and replaced in multiple planthopper clades, subsequent acquisition of another related Bacteroidota symbiont has not been reported [76,77]. In contrast, we identified newly acquired Bacteroidota lineages that were phylogenetically associated with symbiont lineages previously lost from the same scale insect clades. This pattern raises the possibility that host compatibility or predisposition toward particular Bacteroidota lineages may persist after the loss of an ancestral symbiont, facilitating the subsequent establishment of related Bacteroidota. Thus, rather than representing a single ancient Bacteroidota association that was continuously maintained, scale insect-Bacteroidota symbioses may have been repeatedly reconstructed through the acquisition and replacement of related bacterial lineages. Alternatively, the repeated occurrence of related Bacteroidota lineages in distantly related scale insect clades was previously proposed as evidence for host switching based on the phylogenetic similarity of Bacteroidota symbionts between deep-branching scale insects (archeococcoids) and more recently diverged lineages (neococcoids) [25]. However, we consider repeated host switching between distantly related host lineages lacking a recent common ancestor to be less likely than recurrent independent acquisition. The variable genomic states of scale insect-associated Bacteroidota further support independent symbiotic establishment rather than horizontal transmission of established symbionts among unrelated hosts. We therefore propose that the phylogenetic similarity among Bacteroidota symbionts across scale insect clades more likely reflects recurrent recruitment from related bacterial lineages than widespread host switching. The recurrent recruitment of Bacteroidota may be associated with shifts in the nutritional ecology of scale insects, including transitions between feeding on plant phloem sap and plant cell contents during their evolution [78,79]. The nutritional imbalance of phloem sap often necessitates supplementation via microbial symbiosis, whereas parenchymal cell contents provide a more balanced nutrient supply. Such shifts in feeding habits may therefore alter the selective importance of microbial symbiosis, potentially promoting symbiont loss when microbial supplementation becomes less essential and subsequent acquisition of new symbionts when nutritional demands increase again. Broader sampling of host and symbiont diversity, particularly including free-living or facultative relatives of present-day symbionts, will be necessary to identify the ecological sources and evolutionary mechanisms underlying these recurrent acquisitions.

### Long-term symbioses are associated with highly organized symbiotic organs

Our study revealed marked variation in Bacteroidota symbiont localization and bacteriocyte organization among scale insect lineages (Fig. 4; Supplementary Table 5). More highly reduced Bacteroidota symbionts, such as *Walczuchella*, *Conchaspibacter*, and *Cerorastrobacter*, tended to be associated with compact and highly organized bacteriocyte clusters, whereas symbionts with larger genomes were either extracellularly localized or intracellularly distributed within dispersed fat body cells or bacteriocytes. A similar pattern has been observed across diverse hemipteran host clades harboring ancient, genome-reduced symbionts, which commonly possess well-defined bacteriomes arranged as paired or bilaterally symmetrical organs [80–84]. The repeated occurrence of compact bacteriomes across distantly related host lineages suggests that such structure may have evolved repeatedly in highly integrated nutritional symbioses and may be associated with long-term host-symbiont associations. Among the bacteriome architectures observed in this study, a single, compact bacteriome centrally located adjacent to the midgut may represent a near-final form of bacteriocyte arrangement. This architecture was observed in the host of *Cerorastrobacter*, which possesses the most reduced genome among the Bacteroidota symbionts examined here (Figs 2 and 4C). A similar bacteriome architecture occurs in related mealybug lineages harboring the ancient betaproteobacterial symbiont *Tremblaya* (Fig. 1) [85], suggesting that this organization may have either been inherited from a common ancestor or evolved convergently. Such organization may reflect tighter host control over symbiont maintenance and nutrient exchange, potentially indicating a high degree of host–symbiont integration. However, this pattern is not universal, and bacteriome evolution is unlikely to follow a single trajectory. Some hosts harboring ancient, genome-reduced Bacteroidota symbionts, including *Uzinuria* in armored scales and *Blattabacterium* in cockroaches, retain dispersed bacteriocytes, some of which are associated with fat body cells [30,86]. These exceptions suggest that bacteriome organization have evolved along multiple trajectories influenced by lineage-specific developmental, physiological, and ecological constraints. How symbiont replacement influences the development and evolution of bacteriomes remains unclear. Broader taxonomic sampling combined with comparative microscopy will help clarify the evolution of bacteriomes across scale insects and other insect lineages.

### Bacteroidota symbionts exhibit parallel genome reduction

Our results indicate that multiple lineages of Bacteroidota symbionts from scale insects have undergone parallel genome reduction, with repeated losses of genes involved in diverse cellular functions (Fig. 3). Despite their independent evolutionary histories in distinct host lineages, these symbionts show substantial overlap in the functional categories of genes that have been lost, consistent with patterns observed in other reduced symbiont genomes [2,87]. This phenomenon may be attributed to the gradual elimination of redundant genes under relaxed purifying selection during long-term endosymbiosis with a certain host lineage [2,88]. We observed particularly extensive losses of genes involved in DNA replication, recombination, and repair (Fig. 5B), potentially contributing to increased mutation accumulation and genome instability [89,90]. The missing cellular functions may be complemented by either cosymbionts or the host. For example, *Cerorastrobacter* has lost genes involved in DNA repair and DNA replication that are retained in its cosymbiont *Sodalis rastrocola*, providing a potential example of functional complementation similar to that observed for AA biosynthesis (Fig. 6E). Likewise, the extremely reduced *Vidania* genomes (~50 Kbp), which retain only two DNA replication genes, *dnaE* and *dnaQ*, raise the possibility that the missing components of DNA replication are increasingly outsourced to the host or cosymbiont [76]. Together, these patterns suggest that parallel genome reduction is likely accompanied by increasing functional interdependence among symbiotic partners, although the molecular and cellular mechanisms underlying such complementation remain unclear.

Despite these common patterns of genome reduction, Bacteroidota symbionts also exhibited lineage-specific gene retention. Of the 114 genes present in all the analyzed genomes, 93 remain intact without pseudogenization, although some showed evidence of relaxed purifying selection (Fig. 5A). These conserved genes are mainly related to genetic information processing and AA biosynthesis (Supplementary Fig. 8), highlighting the potential significance of these functions for the symbiont cellular maintenance and nutrient provisioning to the insect host, as observed in other sap-feeding insect symbionts (e.g. *Karelsulcia* and *Skilesia*; Supplementary Fig. 9). Genes encoding key components of genetic information processing, including DNA polymerase III subunit α, RNA polymerase subunit α and β, and σ factor, and various ribosomal proteins, remain highly conserved across Bacteroidota symbionts from various insect hosts. Aminoacyl-tRNA synthetase genes for leucine, valine, and lysine, and tRNA modification genes are also broadly retained. However, some genes that are conserved among scale insect symbionts are not universally conserved across Bacteroidota lineages. *Bostrichicola* and *Skimatogeneran*, for example, have independently lost many AA biosynthesis genes that are highly conserved among Bacteroidota symbionts in scale insects (Supplementary Fig. 9). In *Skimatogenerans*, most retained amino-acid biosynthetic capacity is concentrated in the shikimate pathway, potentially reflecting the importance of tyrosine precursors for host cuticle sclerotization and melanization [4]. Thus, although genome reduction has followed broadly similar trajectories across Bacteroidota symbionts, the extent and composition of retained functions have been shaped by the nutritional demands of their respective hosts.

### One Bacteroidota symbiont uses an alternative genetic code and lost protein release factors

One symbiont, *Cerorastrobacter*, with the most degraded genome identified in this study, exhibited a notable modification of the genetic code. It uses genetic code 4, in which UGA encodes tryptophan. Although this code is relatively common in Mycoplasmatales, Entomoplasmatales, and mitochondria [91,92], its occurrence is rare among animal endosymbionts. To date, only six endosymbionts, including this first case in Bacteroidota identified here, have been found to use the genetic code 4 (Table 1). A unique feature of *Cerorastrobacter* is the absence of both *prfA* and *prfB* compared to other symbiont genomes, which at least retain intact *prfA*. This gene encodes RF1, which recognizes TAG and TAA stop codons. Hence, the mechanism by which this symbiont recognizes stop codons during translation remains uncertain. RF1 could potentially be provided by another bacterial symbiont coexisting in the same host or replaced by homologous factors derived from the host or mitochondrion. The CDS of *Cerorastrobacter* are strongly biased toward TAA as a stop codon (Fig. 7D), raising the possibility that the loss of *prfA* is associated with changes in stop-codon usage under strong AT mutational bias. Together, these features suggest remodeling of the translation system in this highly reduced Bacteroidota symbiont, potentially reflecting an evolutionary response to the severe AT mutation bias that increasingly leads to early stop codons and further gene fragmentation.

### What does the frequency of Bacteroidota becoming long-term endosymbionts imply for the origin of organelles?

Our results demonstrate the flexibility of Bacteroidota to repeatedly transition into insect endosymbionts, as well as the apparent preference of scale insects for symbionts from this particular bacterial clade, via a combination of sequencing and imaging data. We highlight the parallel and independent symbiont genome reduction processes together with changes in cell biology and localization, ultimately culminating in a highly specialized host organ near the midgut. The frequency with which the functionally almost identical scale insect symbioses originate and replace each other is particularly striking. Due to the rich taxon sampling of scale insects and the younger age of their symbioses (< 300 Mya) when compared to mitochondria and plastids, much more fine-grained genomics and phylogenomics analyses are feasible in this system than for organelles. The data resulting from our analyses support hypotheses such as the shopping bag model [93], which proposes that organelles originated through successive transient endosymbioses involving multiple bacterial partners, before a stable and permanent integration. We highlight that recurrent symbiont replacements are not necessarily drawn from a broad diversity of bacterial lineages but may instead preferentially involve phylogenetically closely related taxa, as observed here for Bacteroidota. A comparable pattern has also been documented in other insect symbioses involving Pseudomonadota, particularly members of the *Sodalis* lineage [77,94–96]. After convergently evolving for hundreds of millions of years, the symbiont genomes and cell biology are seemingly similar. The phylogenetic signal is blurred by their extreme reduction and limited by the availability of free-living relatives. Inferring the exact number of independent origins and replacements is thus more likely to lead to underestimates rather than overestimates, which should serve as a cautionary tale for future refinements of the shopping bag-related hypotheses concerning organelle evolution.

## Supplementary Material

Supplementary_material_wrag259_revised

## Data Availability

Genome assemblies of the hosts and their Bacteroidota and *Sodalis* symbionts, as well as all Illumina raw sequencing reads, are available in the NCBI BioProject under accession number PRJNA1129605. Other raw data generated in this study are available from the Figshare repository (doi:10.6084/m9.figshare.31017400). All commands used for the bioinformatics analyses are available from the following GitHub repository: https://github.com/ECBSU/ECBSU_manuscripts_code/tree/main/Scale_Insects_Bacteroidota.
