# Supplementary material for: Parallel evolution of Bacteroidota as long-term endosymbionts of insects
Source: ISME J. 2026 Oct 9;20(1):wrag259. doi: 10.1093/ismejo/wrag259 (PMC13712303; doi:10.1093/ismejo/wrag259)
Supplement: Supplementary file 1 [file supplementary_material_wrag259_revised.zip › Supplementary_Figures.pdf]

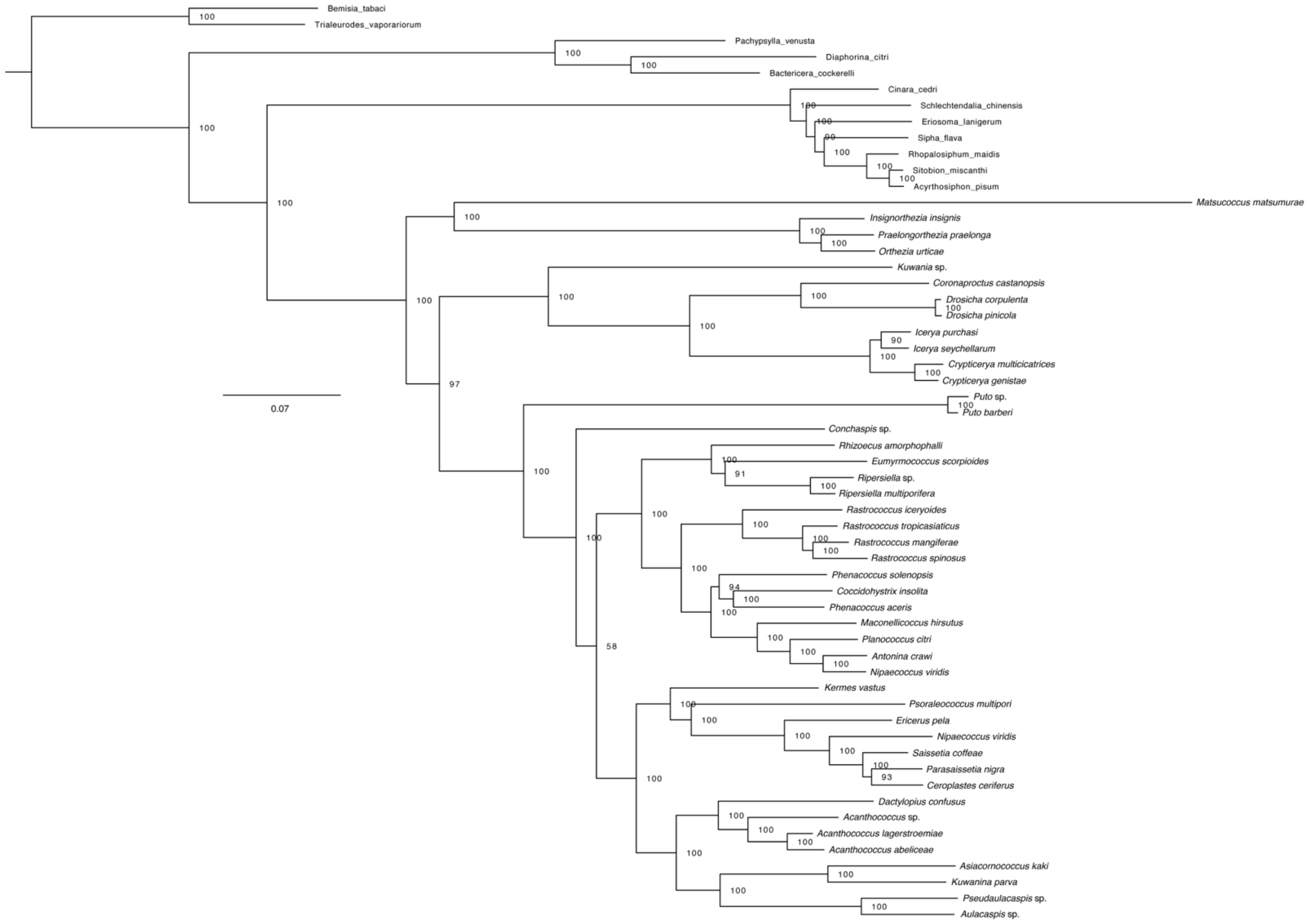

Supplementary Figure 1. Maximum likelihood analysis of scale insect hosts inferred by IQ-TREE from 683 BUSCO genes. Numbers at each node indicate ultrafast bootstrap values.

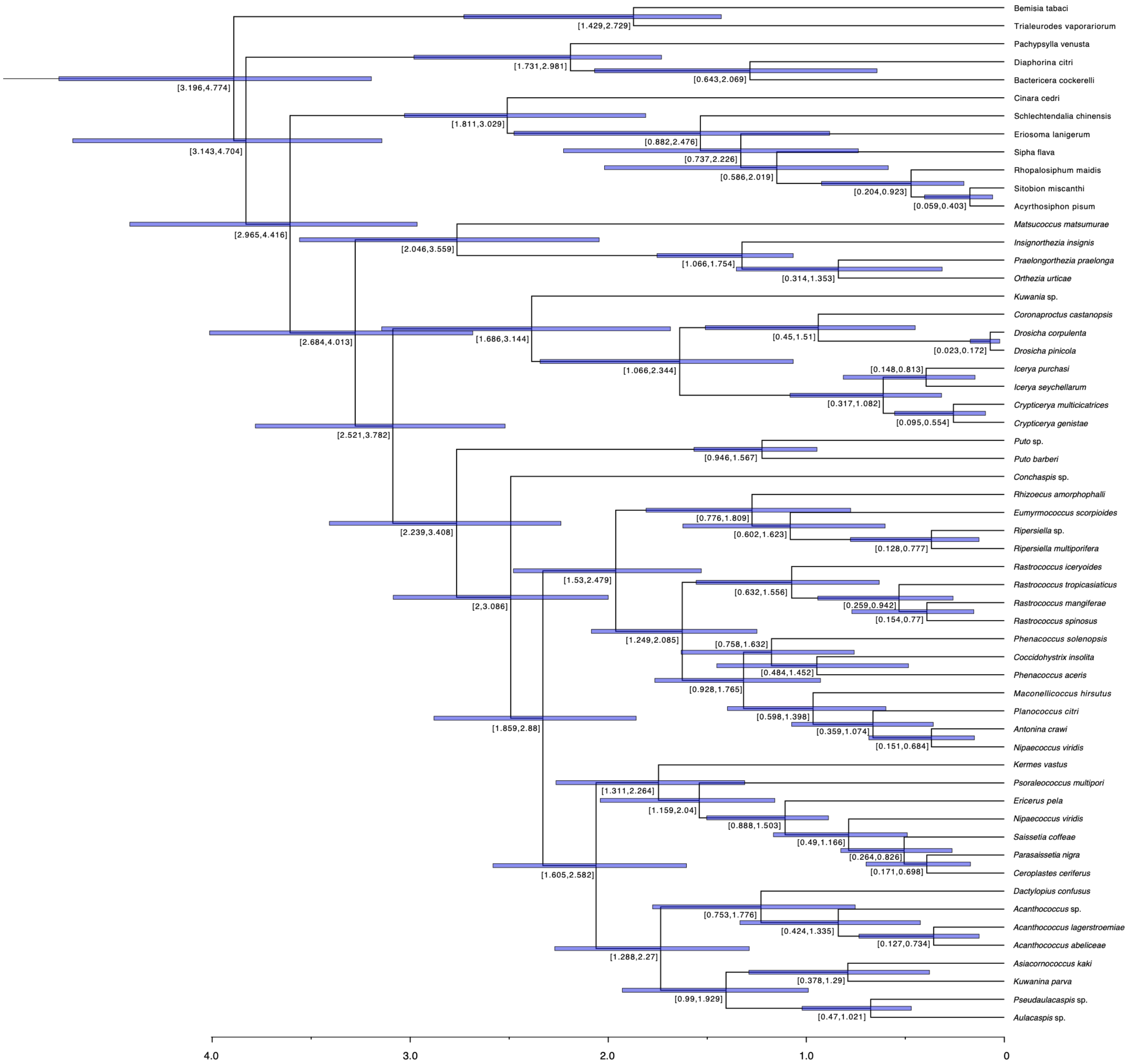

Supplementary Figure 2. Divergence time estimates of scale insect hosts inferred by MCMCtree under the independent rate model. Numbers at each node indicate the estimated range of node age, with 95% confidence intervals displayed by blue bars. Time scale is in units of 100 million years.

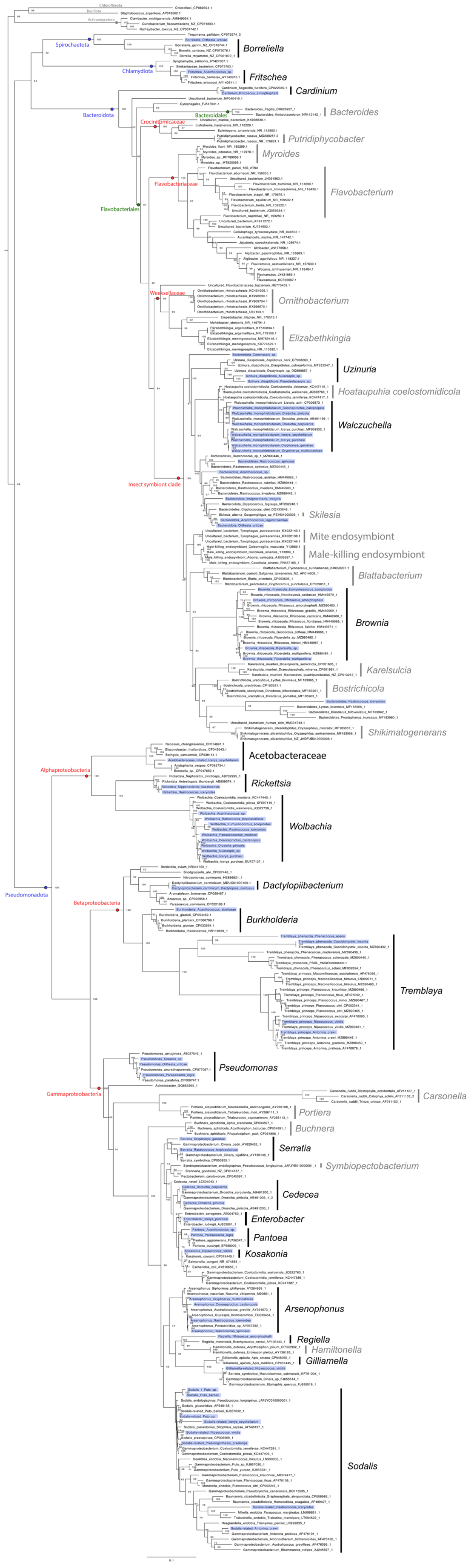

Supplementary Figure 3. Maximum likelihood analysis of bacterial symbionts of scale insects inferred by IQ-TREE from 16S rRNA and 23S rRNA gene sequences. Numbers at each node indicate ultrafast bootstrap values. Symbionts from metagenomes used in this study are highlighted in blue.

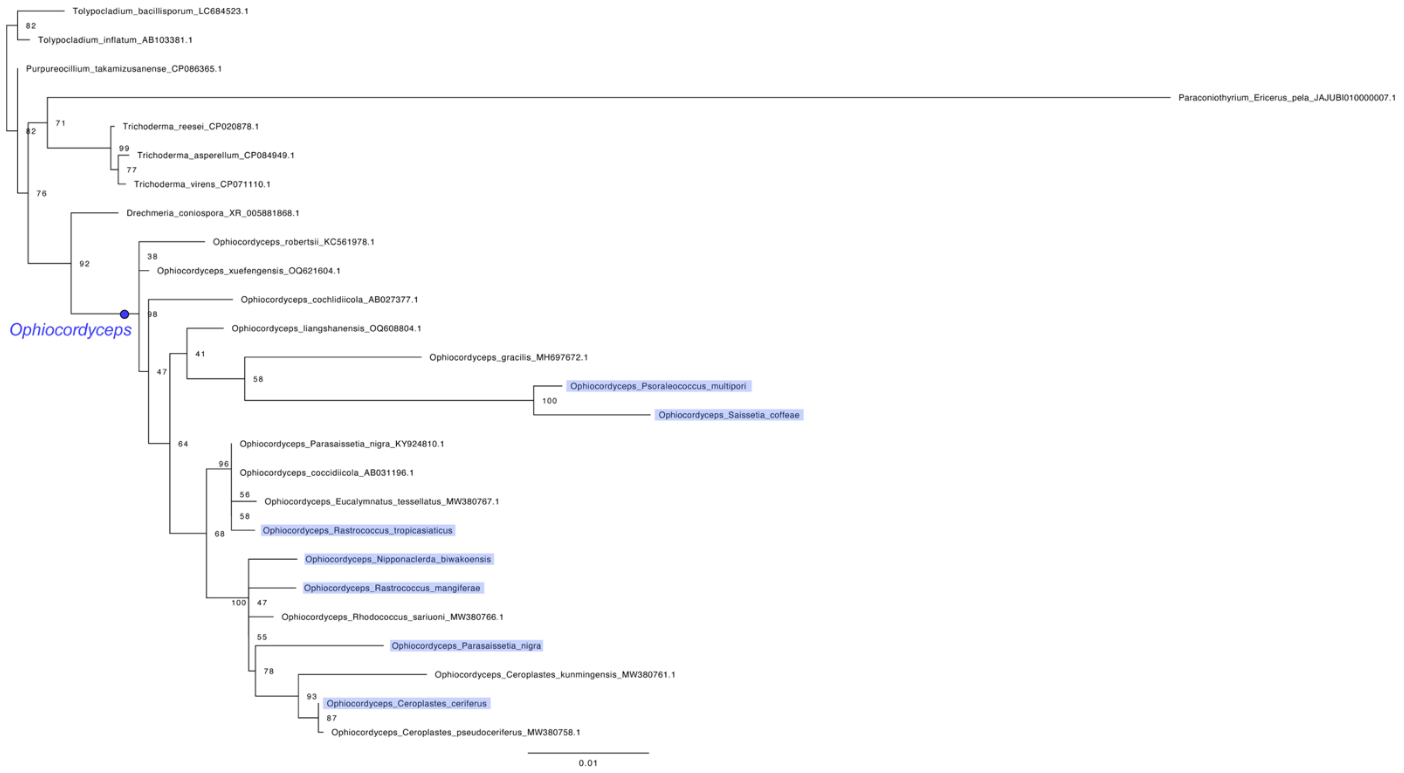

Supplementary Figure 4. Maximum likelihood analysis of fungal symbionts of scale insects inferred by IQ-TREE from 18S rRNA and 28S rRNA gene sequences. Numbers at each node indicate ultrafast bootstrap values. Symbionts from metagenomes used in this study are highlighted in blue.

Remarks. Fungal 18S and 28S sequences extracted from the metagenome of Kermes vastus (KEVA) were excluded from the phylogenetic analysis because they could not be reliably aligned with the other sequences. BLASTn searches indicated that these sequences were most closely related to fungi in the genera *Hirsutella* or *Ophiocordyceps*.

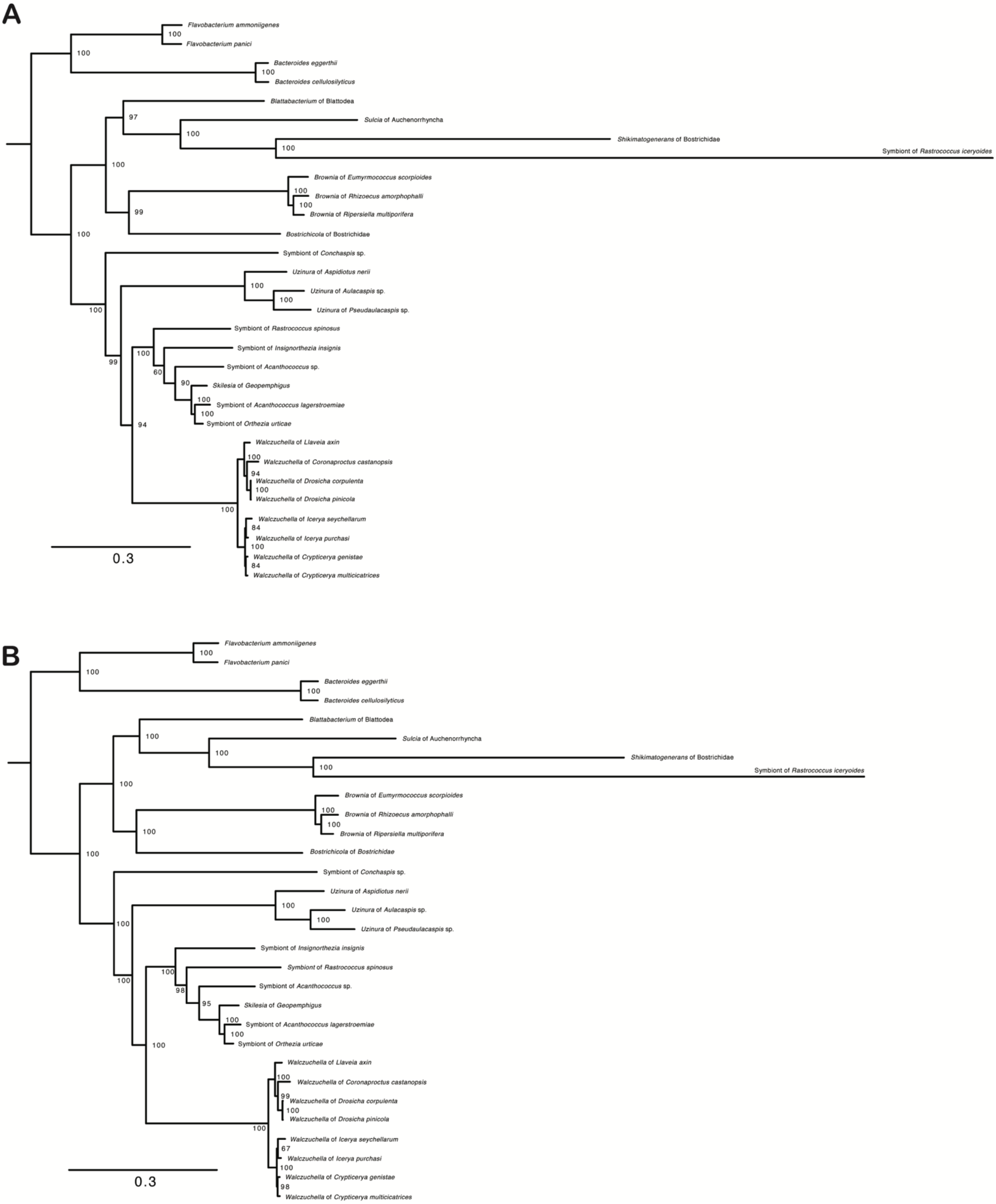

Supplementary Figure 5. Maximum likelihood analyses of Bacteroidota symbionts from scale insects. The phylogenies were reconstructed by IQ-TREE from single-copy orthologous genes sampled with OrthoFinder (65 genes) **(A)** and BUSCO (118 genes) **(B)** under the substitution models, LG+F+R5 and JTTDCMut+F+R5, respectively. Numbers at each node indicate ultrafast bootstrap support values.

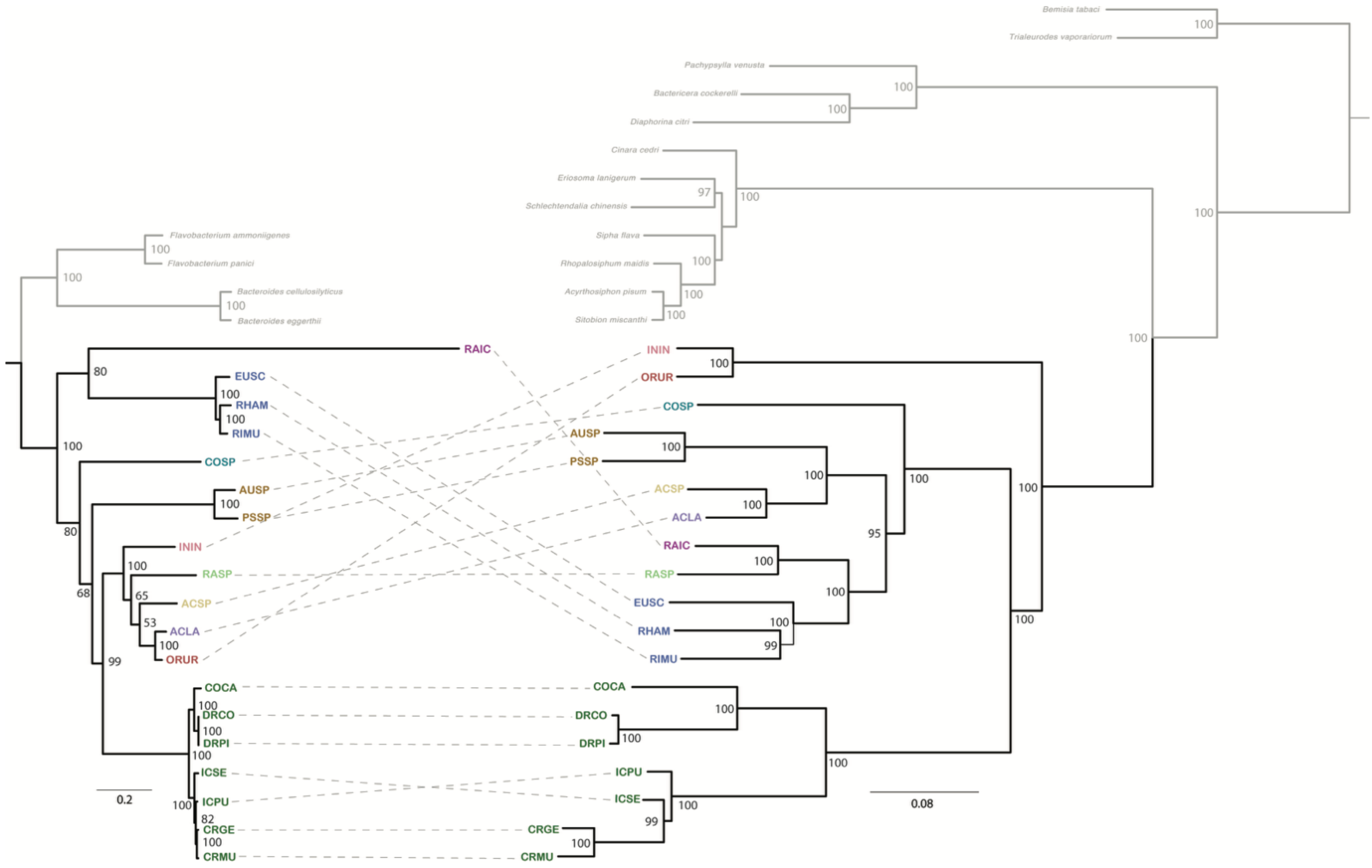

Supplementary Figure 6. Co-phylogeny of Bacteroidota symbionts (left) and their scale insect hosts (right). The symbiont phylogeny was inferred from 84 single-copy orthologous genes identified by OrthoFinder. The host phylogeny was inferred from 1282 BUSCO genes. Both phylogenies were reconstructed using IQ-TREE under the JTTDCMut+F+I+G4 and LG+F+R5 substitution models for the symbionts and host datasets, respectively. Numbers at each node indicate ultrafast bootstrap values. Symbionts are connected by dashed lines to their respective host insects. Outgroup taxa are shown in gray.

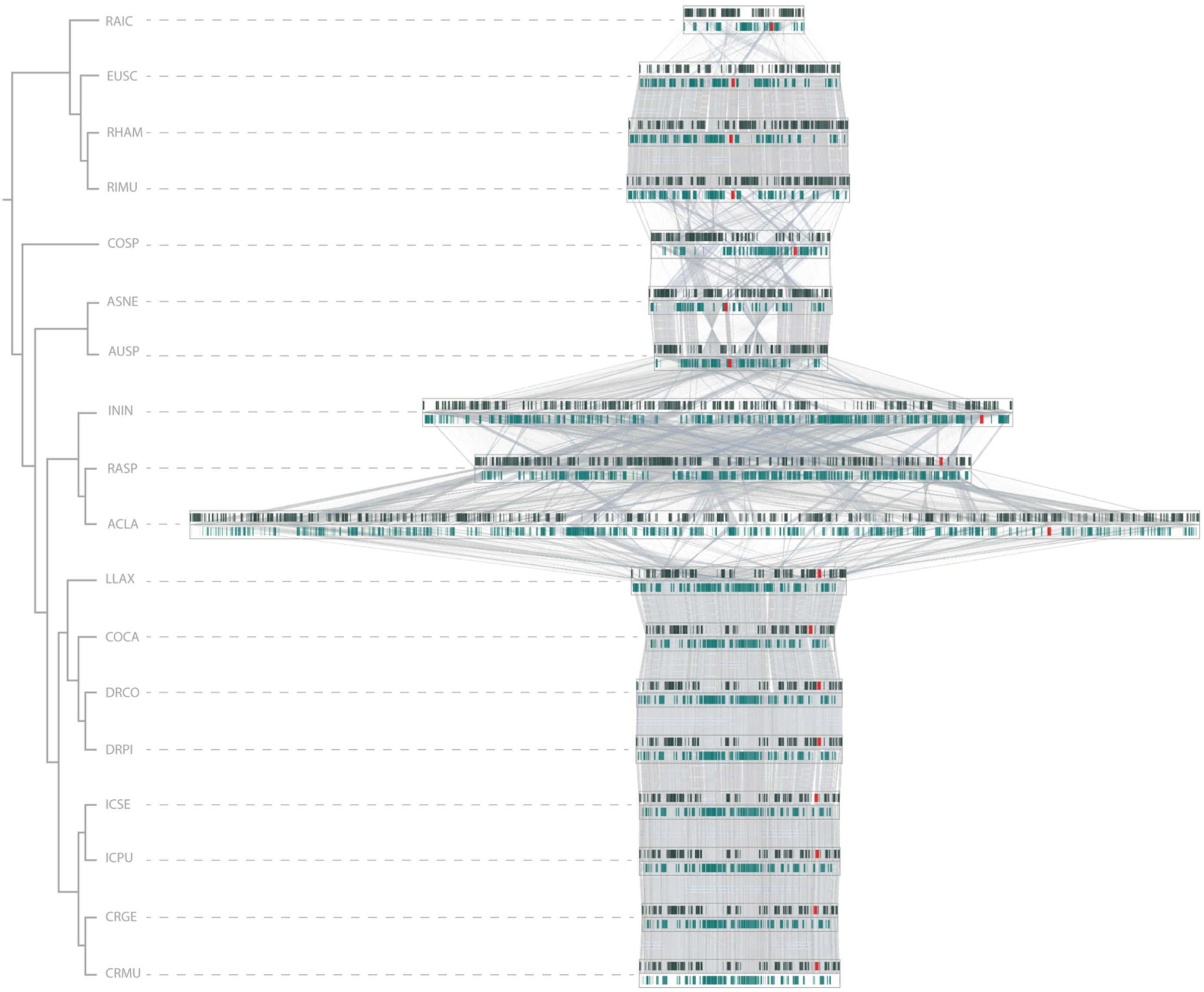

Supplementary Figure 7. Linear genome alignments of Bacteroidota with lines linking homologous genes. Whereas the genes of the same species are largely collinear, significant genomic rearrangements were estimated among different lineages. The genes are color-coded as follows: CDSs on the forward strand are depicted in dark grey, those on the reverse strand in cyan, rRNA in red, tRNA in orange-red, and miscellaneous RNAs in magenta.

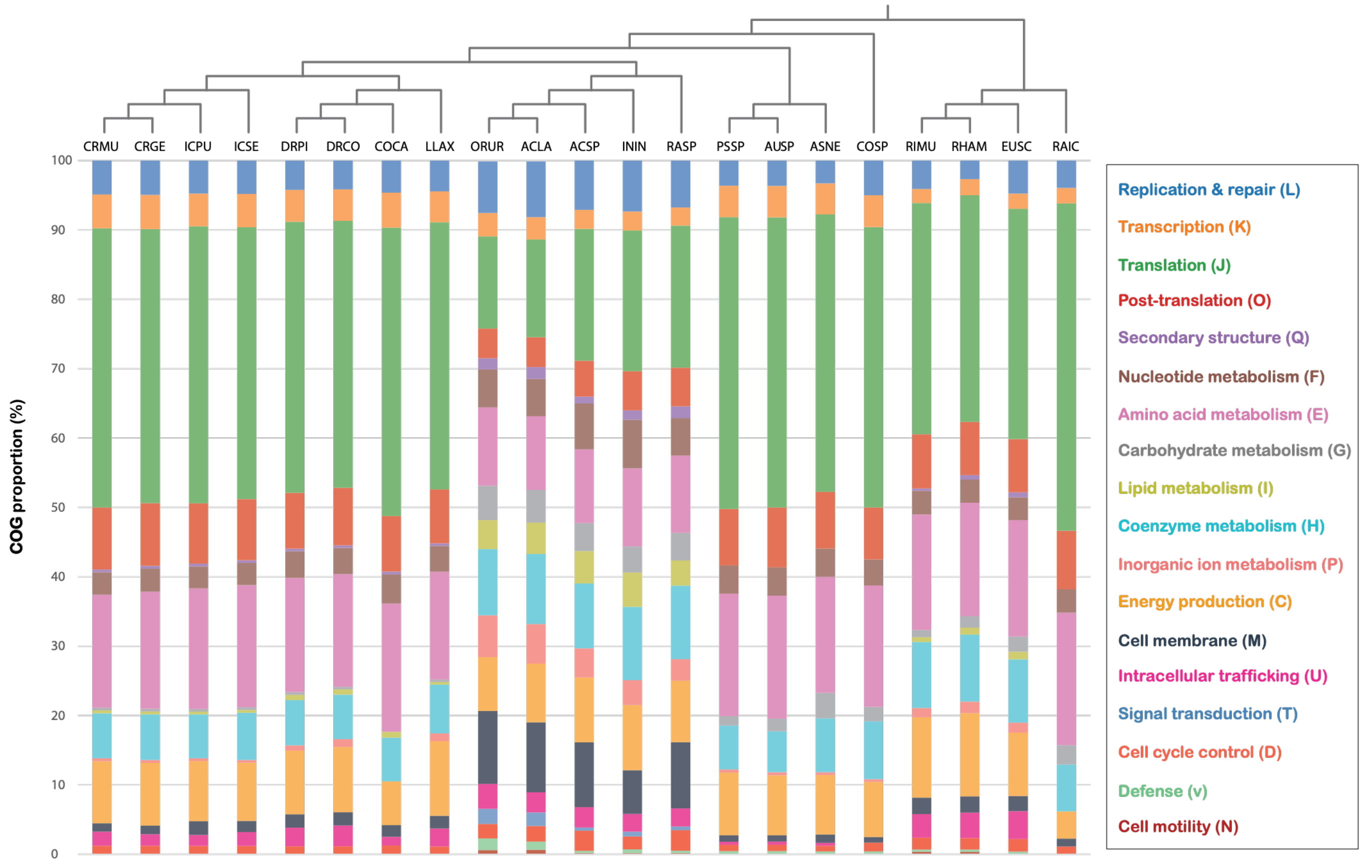

Supplementary Figure 8. Comparison of the clusters of orthologous genes (COG) among Bacteroidota symbionts from scale insects. Color coding is shown in the legend on the right.

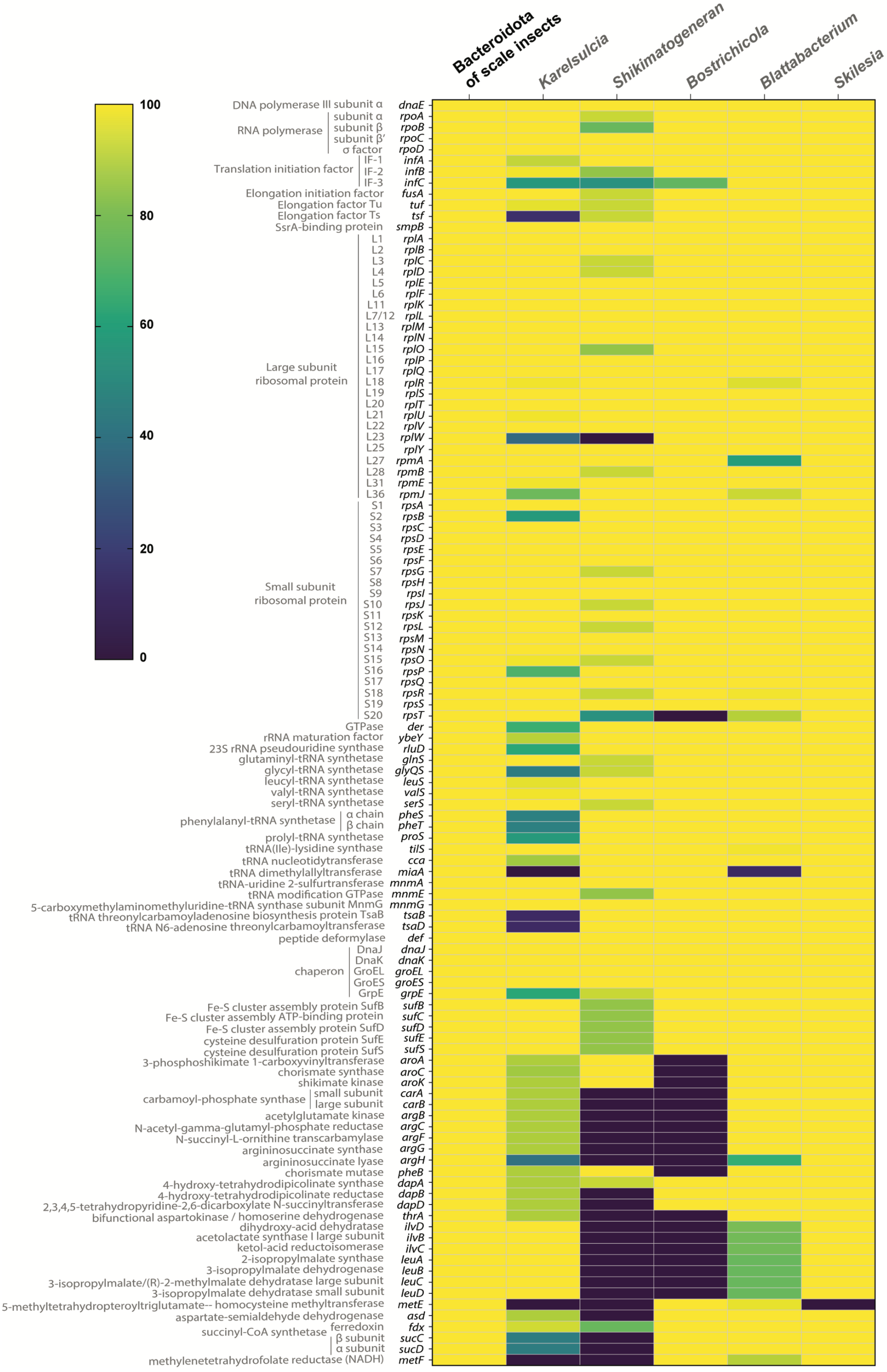

Supplementary Figure 9. Heatmap showing the retention proportions of 114 genes conserved among scale insect symbionts across insect-associated Bacteroidota symbionts. Complete genomes available for *Blattabacterium* (#69), *Bostrichicola* (#4), *Shikimatogenerans* (#13), *Skilesia* (#1), and *Karelsulcia* (#62) were included in the analysis. Gene names, encoded proteins, and associated functions are indicated on the left.

Remarks. Some genes conserved among scale insect symbionts are also retained in other insect-associated Bacteroidota symbionts. However, many of these genes have been independently lost in other symbiont lineages, particularly in the small-genome symbionts *Bostrichicola*, *Shikimatogenerans*, and *Karelsulcia* (142–347 kb). Among genes involved in DNA replication, transcription, and translation, *dnaE* and *rpoCD* are retained in all genomes examined, whereas *infC* is absent from some genomes of the three small-genome symbionts. In addition, *tsf* is absent from most *Karelsulcia* genomes. Most genes involved in ribosome biogenesis are relatively well retained in *Blattabacterium*, *Bostrichicola*, and *Skilesia*, whereas *Shikimatogenerans* and *Karelsulcia* have lost several ribosome biogenesis genes. In particular, *rplW* is absent from all *Shikimatogenerans* genomes. Among genes involved in tRNA modification and biosynthesis, *mnmAG* is fully conserved across all insect-associated Bacteroidota symbionts examined. In contrast, *miaA* and *tsaBD* are absent from almost all *Karelsulcia* genomes, which have also lost several other tRNA-related genes. Most *Blattabacterium* genomes have also lost *miaA*. Among genes involved in post-translational processes, *smpB*, which functions in ribosome rescue, and the protein maturation-related genes *def*, *dnaJK*, and *groLS* are retained across all genomes examined. The iron-sulfur cluster biosynthesis genes *sufBCDE* are also conserved across most symbionts but are absent from some *Shikimatogenerans* genomes. Amino acid biosynthesis genes are substantially reduced in *Bostrichicola* and *Shikimatogenerans*. *Karelsulcia* retains genes involved in cysteine, (iso)leucine, and valine biosynthesis (*ilvBCD*, *leuABCD*, and *sufS*), although some genomes have lost additional amino acid biosynthesis genes. In contrast, some *Blattabacterium* genomes have specifically lost genes involved in (iso)leucine and valine biosynthesis, as well as *argH*. Genes involved in energy production are relatively conserved in *Bostrichicola*, *Blattabacterium*, and *Skilesia*, whereas *sucCD* and *metF* are absent from *Shikimatogenerans* and some *Karelsulcia* genomes. *Skilesia* retains nearly all genes conserved among scale insect symbionts, with *metE* being the notable exception.
